# Direct Identification of the Continuous Relaxation Time and Frequency Spectra of Viscoelastic Materials

**DOI:** 10.3390/ma17194870

**Published:** 2024-10-03

**Authors:** Anna Stankiewicz

**Affiliations:** Department of Technology Fundamentals, Faculty of Production Engineering, University of Life Sciences in Lublin, 20-612 Lublin, Poland; anna.m.stankiewicz@gmail.com

**Keywords:** viscoelasticity, relaxation spectra, linear relaxation modulus, direct spectrum approximation, identification algorithm, model integral square error, noise robustness

## Abstract

Relaxation time and frequency spectra are not directly available by measurement. To determine them, an ill-posed inverse problem must be solved based on relaxation stress or oscillatory shear relaxation data. Therefore, the quality of spectra models has only been assessed indirectly by examining the fit of the experiment data to the relaxation modulus or dynamic moduli models. As the measures of data fitting, the mean sum of the moduli square errors were usually used, the minimization of which was an essential step of the identification algorithms. The aim of this paper was to determine a relaxation spectrum model that best approximates the real unknown spectrum in a direct manner. It was assumed that discrete-time noise-corrupted measurements of a relaxation modulus obtained in the stress relaxation experiment are available for identification. A modified relaxation frequency spectrum was defined as a quotient of the real relaxation spectrum and relaxation frequency and expanded into a series of linearly independent exponential functions that are known to constitute a basis of the space of square-integrable functions. The spectrum model, given by a finite series of these basis functions, was assumed. An integral-square error between the real unknown modified spectrum and the spectrum model was taken as a measure of the model quality. This index was proved to be expressed in terms of the measurable relaxation modulus at uniquely defined sampling instants. Next, an empirical identification index was introduced in which the values of the real relaxation modulus are replaced by their noisy measurements. The identification consists of determining the spectrum model that minimizes this empirical index. Tikhonov regularization was applied to guarantee model smoothness and noise robustness. A simple analytical formula was derived to calculate the optimal model parameters and expressed in terms of the singular value decomposition. A complete identification algorithm was developed. The analysis of the model smoothness and model accuracy for noisy measurements was carried out. The equivalence of the direct identification of the relaxation frequency and time spectra has been demonstrated when the time spectrum is modeled by a series of functions given by the product of the relaxation frequency and its exponential function. The direct identification concept can be applied to both viscoelastic fluids and solids; however, some limitations to its applicability have been pointed out. Numerical studies have shown that the proposed identification algorithm can be successfully used to identify Gaussian-like and Kohlrausch–Williams–Watt relaxation spectra. The applicability of this approach to determining other commonly used classes of relaxation spectra was also examined.

## 1. Introduction

Although the first papers concerning relaxation time and frequency spectra determination come from the late 1940s of the 20th century [1,2], the recovery of the relaxation spectrum from the measurement data is still an active area of research in rheology and the identification of time-variable viscoelastic mechanical characteristics [3,4,5,6,7,8,9,10]. Relaxation time and frequency spectra, with no direct accessible measurements, are recovered from the stress relaxation or oscillatory shear data by applying appropriate identification methods intended for determination of the spectra. The relaxation spectrum identification task is the problem of numerically solving a system of Fredholm integral equations of the first kind obtained for discrete measurements of the relaxation modulus or storage and loss modulus data. These problems are well-known to be the ill-posed inverse problems, the solutions to which, if any, are very sensitive to even small changes in the experiment data leading to arbitrarily large changes in the determined relaxation spectrum. Therefore, special stable algorithms are requisite to determine noise-robust relaxation spectrum models.

Over the last 80 years, different analytical and numerical tools have been applied to identify the relaxation spectrum. Numerous classes of algorithms were developed to determine continuous and discrete relaxation spectra models. Many theoretical papers have been devoted to the methods and algorithms for relaxation spectra determination, e.g., see [3,6,9,11,12,13,14]. In addition, experimental studies conducted for various viscoelastic materials motivated relaxation spectra models and appropriate identification algorithms, for example as seen in [4,15,16,17,18]. Reviews of these methods and algorithms can be found in many papers, for example [5,19,20] and, most recently, in [10,18].

After a few models and algorithms were derived from an application of the Post–Widder differential formula [21,22,23], many more intricate methods and models have been obtained based on the usage of the least-squares identification applied both to the relaxation modulus measurements obtained in the stress relaxation test [7,10,24,25,26,27,28] and to the measurements of the storage and loss moduli resulted from the oscillatory shear experiment [3,4,5,6,8,11,12,14,15,16,17,18]. For example, in [26,27,28], different identification algorithms were derived for the optimal regularized least-squares identification of relaxation time and frequency spectra in the classes of models defined by a finite series of different basis functions. In consequence, the quality of the spectra models was estimated by the mean sum of relaxation modulus or dynamic moduli square errors used as a measure, the minimization of which, with or without regularization, was an essential step of the identification algorithm. In some papers, e.g., [4,7,29], the pure least-squares identification was applied, while for example in [3,5,11,26,27,28] the regularized least-squares were used with various rules applied for the choice of regularization parameters to ensure the stability of the scheme and model smoothness. Recently, in [10], the best smoothed spectrum model—which reproduces the relaxation modulus measurements with a small error of the relaxation modulus model by minimizing the integral square norm of the spectrum—was found; however, here, the identification criterion is related only to the spectrum model and not to the unknown real spectrum, and the model error is assessed in terms of the measurement-available relaxation modulus. 

In this paper, a new approach is proposed based on direct approximation of the real unknown relaxation time spectrum by a series of appropriately selected basis functions. It was assumed that discrete-time noise-corrupted measurements of a relaxation modulus obtained in the stress relaxation experiment are available for identification. First, a modified relaxation frequency spectrum was defined as a quotient of the real relaxation spectrum and relaxation frequency. This spectrum was expanded into a series of exponential functions forming a basis of the space of square-integrable functions [30]. Such expansion is equivalent to the expansion of the relaxation time spectrum into a series of basis functions, these being the products of the relaxation frequency and the exponential function of it. The spectra models, given by the finite series of these basis functions, were assumed. An integral square error between the real unknown spectrum and the spectrum model was taken as a measure of the model quality index. The equivalence of such defined indices for the relaxation time spectrum and the modified frequency spectrum was proved, which means an equivalence between the respective spectra approximation tasks. Next, an empirical identification index was introduced by replacing the real relaxation modulus by their noise measurements. The resulting identification problem is a linear-quadratic optimization task in which Tikhonov regularization is applied to ensure its well-posedness. Simple analytical formula for determining the optimal model parameters was derived; the singular value decomposition can be used for algebraic computations. A complete identification algorithm for determining the optimal models of the relaxation spectra has been developed. Model smoothness and noise robustness were analyzed. The results of simulation studies conducted for uni- and double-mode Gaussian-like and Kohlrausch–Williams–Watts relaxation spectra are presented. Finally, based on the congruence of the boundary conditions of the real spectra and the model basis functions, a short analysis of the applicability of the proposed approach is outlined for different classes of the real spectra, and its limitations are pointed out. It is demonstrated that the concept of direct relaxation spectrum identification can be applied both for viscoelastic fluids and viscoelastic solids. In Appendix A, the proofs and derivations of some mathematical formulas and results are given. 

The idea of using a series expansion of the spectrum model has been previously applied both in the time [26,27,28] and frequency [8] domains; however, in these papers, the identification indices, being minimized, were related to the models of the relaxation or dynamic moduli and not to the unknown spectrum model. Here, the use of appropriately selected basis functions of the relaxation spectrum model allowed for linking the model quality index, related directly to the unknown spectrum, with the relaxation modulus measurements. This means that the identification index being minimized, although expressed in terms of the relaxation modulus measurements, refers directly to the unknown relaxation spectrum, not to the measured relaxation modulus. This new approach is proposed and used in this paper for the first time.

## 2. Materials and Methods

### 2.1. Relaxation Spectra

It is widely assumed in rheology [31,32,33] that the linear relaxation modulus $G\left(t\right)$ (i.e., the stress per unit strain) has a relaxation spectrum representation of the form

(1) $$G\left(t\right)=\int_{0}^{\infty }\frac{H\left(\tau \right)}{\tau }e^{-t/\tau }d\tau ,$$

or equivalently by

(2) $$G\left(t\right)=\int_{0}^{\infty }\frac{H\left(v\right)}{v}e^{-tv}dv,$$

where the relaxation time $H\left(\tau \right)$ and frequency $H\left(v\right)$ spectra, related by

(3) $$H\left(v\right)=H\left(\frac{1}{v}\right),\;H\left(\tau \right)=H\left(\frac{1}{\tau }\right),$$

characterize the distributions of relaxation times τ and frequencies v. They are generalizations of discrete Maxwell spectra [31,32] to continuous functions of τ and v. Although other definitions of the relaxation spectrum are used in the literature, for example, in [34,35,36], the definition introduced by Equations (1) and (2) dominates. 

### 2.2. Models

Following [26], the modified spectrum is introduced

(4) $$H^{M}\left(v\right)=\frac{H\left(v\right)}{v},$$

where the upper index of $H^{M}\left(v\right)$ means “modified”. Model transformation defined by (4) is a bijection. Equation (2) can be rewritten as follows:

(5) $$G\left(t\right)=\int_{0}^{\infty }H^{M}\left(v\right)e^{-tv}dv,$$

i.e., the modulus $G\left(t\right)$ is directly the Laplace integral of the spectrum $H^{M}\left(v\right)$.

Assume that $H^{M}\left(v\right)\in L^{2}\left(0,\right.\left.\infty \right)$, where $L^{2}\left(0,\right.\left.\infty \right)$ is the space of real-valued square-integrable functions on the interval $\left(0,\right.\left.\infty \right)$. The respective sufficient conditions are given by Theorem 3 in [37]. The set of the linearly independent exponential functions $\left\{e^{-\alpha kv}\right\}$, k=0,1,…, where α>0, i.e., the kernel of the Laplace transformation, form a basis of the space $L^{2}\left(0,\right.\left.\infty \right)$ [30]. Thus, the modified relaxation spectrum can be expressed as:

(6) $$H^{M}\left(v\right)=\sum_{k=0}^{\infty }g_{k}h_{k}\left(v\right),$$

with basis functions defined as follows

(7) $$h_{k}\left(v\right)=e^{-\alpha kv},$$

where parameter α>0 is a time-scaling factor expressed in seconds, while $g_{k}$ are constant model parameters.

By (4) and (6), for the real relaxation spectrum of the material we have:

(8) $$H\left(v\right)=\sum_{k=0}^{\infty }g_{k}h_{k}\left(v\right)v.$$

The modified spectrum $H^{M}\left(v\right)\in L^{2}\left(0,\right.\left.\infty \right)$, then, $H^{M}\left(v\right)\to 0$ as v→∞ and the first basis function can be neglected. For practical reasons, it is convenient to replace the infinite summation in the above equation with a finite one of K first terms, i.e., to approximate the relaxation spectrum $H^{M}\left(v\right)$ (4) by a model of the form

(9) $$H_{K}^{M}\left(v\right)=\sum_{k=1}^{K}g_{k}h_{k}\left(v\right),$$

where the lower index of $H_{K}^{M}\left(v\right)$ is the number of model summands. Spectrum $H^{M}\left(v\right)$ (4) is expressed in Pa·s, so also Pa·s is a unit of the model’s parameters $g_{k}$. The model of the original spectrum $H\left(v\right)$ related to (9) takes the form

(10) $$H_{K}\left(v\right)=vH_{K}^{M}\left(v\right)=\sum_{k=1}^{K}g_{k}h_{k}\left(v\right)v.$$

The related relaxation modulus model, by (5) and (9) is described by the following:

(11) $$G_{K}\left(t\right)=\int_{0}^{\infty }H_{K}^{M}\left(v\right)e^{-tv}dv=\sum_{k=1}^{K}g_{k}\frac{1}{t+\alpha k}=\sum_{k=1}^{K}g_{k}\phi_{k}\left(t\right),$$

where the basis functions, expressed in $s^{-1}$, are as follows

(12) $$\phi_{k}\left(t\right)=\frac{1}{t+\alpha k}.$$

By the second equality in (3), (8), and (7), we obtain the following series representation of the relaxation time spectrum

(13) $$H\left(\tau \right)=\sum_{k=0}^{\infty }g_{k}e^{-\frac{\alpha k}{\tau }}\frac{1}{\tau },$$

Omitting, as above, the first component and considering the K next terms of the series (13), the relaxation spectrum $H\left(\tau \right)$ can be approximated by a model of the form

(14) $$H_{K}\left(\tau \right)=\sum_{k=1}^{K}g_{k}h_{k}\left(\tau \right),$$

where the basis functions

(15) $$h_{k}\left(\tau \right)=e^{-\frac{\alpha k}{\tau }}\frac{1}{\tau }$$

and the model parameters $g_{k}$, expressed in Pa·s, are identical to that of model (9). By (1) and (14), the relaxation modulus model is as follows:

$$G_{K}\left(t\right)=\int_{0}^{\infty }\frac{H_{K}\left(\tau \right)}{\tau }e^{-t/\tau }d\tau =\sum_{k=1}^{K}g_{k}\int_{0}^{\infty }\frac{1}{\tau^{2}}e^{-\frac{t+\alpha k}{\tau }}d\tau =\sum_{k=1}^{K}g_{k}\phi_{k}\left(t\right),$$

where the basis functions are given by (12), i.e., it is identical to the model described by (11).

### 2.3. Properties of the Basis Functions

**Figure 1:**
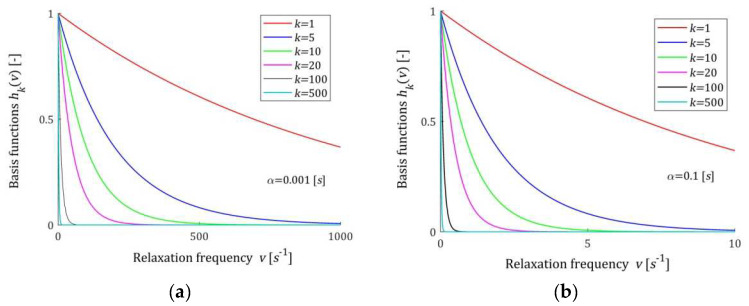
Basis functions $h_{k}\left(v\right)=e^{-\alpha kv}$ (7) of the relaxation spectrum model $H_{K}^{M}\left(v\right)$ (9) for two time-scaling factors, α: (**a**) α=0.001 [s]; (**b**) α=0.1 [s]; k=1, 5, 10, 20, 100, 500.

**Figure 2:**
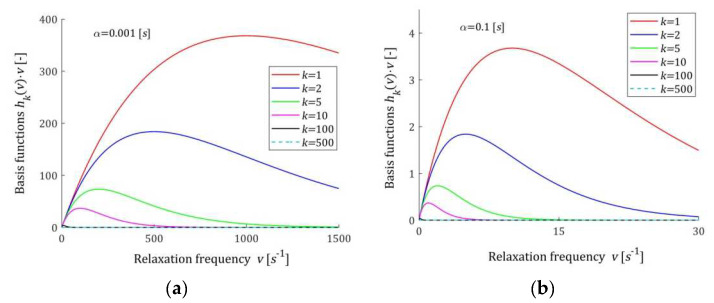
Basis functions $h_{k}\left(v\right)v$ of the relaxation spectrum model $H_{K}\left(v\right)$ (10) for two time-scaling factors, α: (**a**) α=0.001 [s]; (**b**) α=0.1 [s]; k=1, 2, 5, 10, 100, 500.

**Figure 3:**
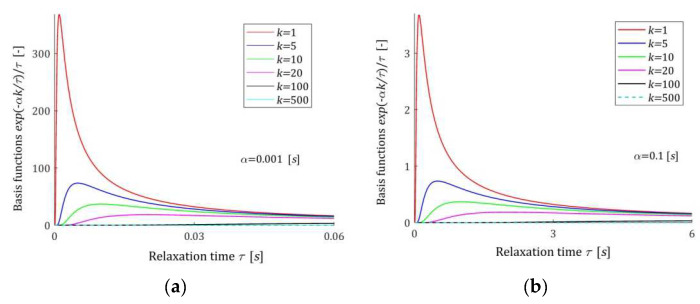
Basis functions $h\left(\tau \right)=e^{-\frac{\alpha k}{\tau }}/\tau$ (15) of the relaxation time spectrum model $H_{K}\left(\tau \right)$ (14) for two time-scaling factors, α: (**a**) α=0.001 [s]; (**b**) α=0.1 [s]; k=1, 5, 10, 20, 100, 500.

A few basis functions $h_{k}\left(v\right)$ (7) of the model $H_{K}^{M}\left(v\right)$ (9) are shown in Figure 1 for two different values of the time-scale factor α, while in Figure 2, the basis functions $h_{k}\left(v\right)v$ of the model $H_{K}\left(v\right)$ (10) are given. In Figure 3, the basis functions $h_{k}\left(\tau \right)$ (15) of the relaxation time spectrum model $H_{K}\left(\tau \right)$ (14) are demonstrated.

**Figure 4:**
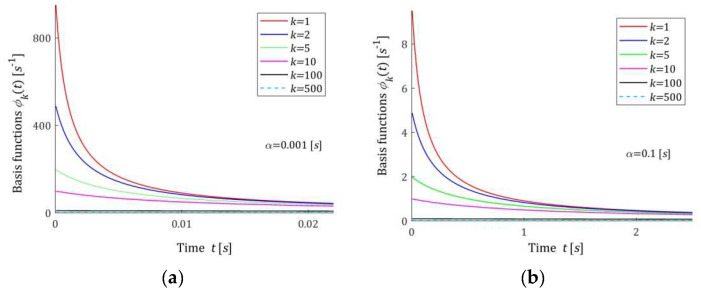
Basis functions $\phi_{k}\left(t\right)$ (12) of the relaxation modulus model $G_{K}\left(t\right)$ (11) for two time-scaling factors, α: (**a**) α=0.001 [s]; (**b**) α=0.1 [s]; k=1, 2, 5, 10, 100, 500.

Figure 4 shows the hyperbolic basis functions $\phi_{k}\left(t\right)$ (12) of the relaxation modulus model $G_{K}\left(t\right)$ (11). Functions $\phi_{k}\left(t\right)$ are almost constant in time and near zero (in the considered time intervals) for k=100 and k=1000. However, for smaller indices k, they are in good agreement with the real relaxation modulus obtained in the stress relaxation test.

The basis functions of the models $H_{K}^{M}\left(v\right)$, $H_{K}\left(v\right)$, and $G_{K}\left(t\right)$ are positive definite. Functions $h_{k}\left(v\right)$ (7) are monotonically decreasing, while basis functions $h_{k}\left(v\right)v$ of the model $H_{K}\left(v\right)$ (10) have global maxima equal to $1/\left(\alpha ke\right)$ for the relaxation frequencies $v=1/\left(\alpha k\right)$. In addition, basis functions $h_{k}\left(\tau \right)$ (15) have unique maxima equal to $1/\left(\alpha ke\right)$ at the relaxation times τ=αk. Relaxation modulus basis functions $\phi_{k}\left(t\right)$ (12) are monotonically decreasing. For large v, and in particular for v→∞, the basis functions $h_{k}\left(v\right)$ and $h_{k}\left(v\right)v$ decrease exponentially to zero. Similarly, for τ→∞ functions $h_{k}\left(\tau \right)\to 0$, faster than the exponential function. Functions $\phi_{k}\left(t\right)$ tends to zero hyperbolically as t→∞. 

## 3. Results

In this section, the problem of optimal spectrum approximation in the class of models defined by a finite series of the introduced basis functions is formulated and solved. First, it is demonstrated that the problem of the optimal—in the sense of an integral square error—approximation of the modified relaxation frequency spectrum is equivalent to the problem of the optimal approximation of the relaxation time spectrum. It is also proved that, due to the choice of exponential basis functions, the integral square model error can be expressed in terms of the relaxation modulus in the sampling points uniquely determined by the basis functions of the spectrum model. Next, an empirical identification index is introduced in which the values of the real relaxation modulus are replaced by their noisy, in general, measurements. The optimal models of the relaxation time and frequency spectra are determined by solving the linear-quadratic identification task. However, this problem turned out to be ill-conditioned. Therefore, Tikhonov regularization is applied resulting in the stable, noise-robust and simple identification rule. Next, the equations and functions essential for the proposed identification scheme are described in terms of the singular value decomposition of the basic matrix of this identification problem. Model smoothness is estimated, error of the relaxation modulus is evaluated, noise robustness and convergence analysis is conducted. The complete identification algorithm is presented. The results of simulation studies for Gaussian-like and Kohlrausch–Williams–Watts relaxation spectra describing many real materials are presented. Finally, based on the congruence of the boundary conditions of the real spectra and the basis functions, a rough applicability analysis of the proposed approach is outlined for different classes of the real spectra. 

### 3.1. Spectrum Approximation

Model $H_{K}^{M}\left(v\right)$ (9) approximates the modified spectrum $H^{M}\left(v\right)$ (4). As a measure of the model (9) accuracy the integral square index is taken

(16) $$J\left(g_{K}\right)=\int_{0}^{\infty }{\left[H^{M}\left(v\right)-H_{K}^{M}\left(v\right)\right]}^{2}dv,$$

where $g_{K}={\left[\begin{array}{ccc} g_{1} & \cdots & g_{K} \end{array}\right]}^{T}$ is an K—element vector of the model (9) parameters; superscript “T” indicates transpose. In view of the additive form of the model (9), index $J\left(g_{K}\right)$ can be expressed as follows:

(17) $$J\left(g_{K}\right)=\int_{0}^{\infty }H^{M}{\left(v\right)}^{2}dv-2\sum_{k=1}^{K}g_{k}\int_{0}^{\infty }H^{M}\left(v\right)h_{k}\left(v\right)dv+\sum_{k=1}^{K}\sum_{m=1}^{K}g_{k}g_{m}\varphi_{km},$$

where, by (7), the coefficients

(18) $$\varphi_{km}=\int_{0}^{\infty }h_{k}\left(v\right)h_{m}\left(v\right)dv=\int_{0}^{\infty }e^{-\alpha \left(k+m\right)v}dv=\frac{1}{\alpha \left(k+m\right)}.$$

Since, in view of (5) and (7), we have:

$$\int_{0}^{\infty }H^{M}\left(v\right)h_{k}\left(v\right)dv=\int_{0}^{\infty }H^{M}e^{-\alpha kv}dv=G\left(\alpha k\right),$$

the above index takes the form

(19) $$J\left(g_{K}\right)=\int_{0}^{\infty }{H^{M}\left(v\right)}^{2}dv-2\sum_{k=1}^{K}g_{k}G\left(\alpha k\right)+\sum_{k=1}^{K}\sum_{m=1}^{K}g_{k}g_{m}\varphi_{km}.$$

Model $H_{K}\left(\tau \right)$ (14) approximates the real spectrum $H\left(\tau \right)$. As a measure of the model (14) accuracy the square index, analogous to (16), is taken

(20) $$J\left(g_{K}\right)=\int_{0}^{\infty }{\left[H\left(\tau \right)-H_{K}\left(\tau \right)\right]}^{2}d\tau ,$$

where $g_{K}$ is a vector of the model (14) parameters. In view of (14) and (15), the above index can be expressed as follows:

(21) $$J\left(g_{K}\right)=\int_{0}^{\infty }H^{2}\left(\tau \right)d\tau -2\sum_{k=1}^{K}g_{k}\int_{0}^{\infty }H\left(\tau \right)h_{k}\left(\tau \right)d\tau +\;\sum_{k=1}^{K}{\sum_{m=1}^{K}g_{k}g}_{m}\int_{0}^{\infty }h_{k}\left(\tau \right)h_{m}\left(\tau \right)d\tau ,$$

where, by (15) and (1)

$$\int_{0}^{\infty }H\left(\tau \right)h_{k}\left(\tau \right)d\tau =\int_{0}^{\infty }H\left(\tau \right)e^{-\frac{\alpha k}{\tau }}\frac{1}{\tau }d\tau =G\left(\alpha k\right),$$

while the integrals

(22) $$\int_{0}^{\infty }h_{k}\left(\tau \right)h_{m}\left(\tau \right)d\tau =\int_{0}^{\infty }{\frac{1}{\tau^{2}}e}^{-\frac{\alpha \left(k+m\right)}{\tau }}d\tau =\frac{1}{\alpha \left(k+m\right)}=\varphi_{km},$$

are identical to that given by (18). Combining the above with (21) yields

$$J\left(g_{K}\right)=\int_{0}^{\infty }H^{2}\left(\tau \right)d\tau -2\sum_{k=1}^{K}g_{k}G\left(\alpha k\right)+\sum_{k=1}^{K}\sum_{m=1}^{K}g_{k}g_{m}\varphi_{km}.$$

For the material relaxation spectra $H\left(\tau \right)$ and $H^{M}\left(v\right)$, by (4) and simple substitution based on (3), we have the following:

(23) $$\int_{0}^{\infty }{H^{M}\left(v\right)}^{2}dv=\int_{0}^{\infty }\frac{{H\left(v\right)}^{2}}{v^{2}}dv=\int_{0}^{\infty }{H\left(\frac{1}{v}\right)}^{2}\frac{1}{v^{2}}dv=\int_{0}^{\infty }{H\left(\tau \right)}^{2}d\tau ,$$

that is, the integral square index defined by (20) is identical to that defined by (16); therefore, the same notation was used.

### 3.2. Identification Problem 

Suppose that a certain identification experiment (stress relaxation test [1,33,38]) performed on the specimen of the material under investigation resulted in a set of measurements of the relaxation modulus $\left\{\bar{G}\left(t_{k}\right)=G\left(t_{k}\right)+z\left(t_{k}\right)\right\}$ at the sampling instants $t_{k}=\alpha k$, k=1,…,K; where $z\left(t_{k}\right)$ is additive measurement noise. Generally, identification consists of selecting within the given class of models, which ensures the best fit to the measurement results. Classically, the mean square identification index related to the measurements of the relaxation modulus $\bar{G}\left(t_{k}\right)$ is used; compare [7,24,26,27,28]. This means that the model quality index is not related directly to the unknown relaxation spectrum, which is inaccessible by measurement, but to the measurement-available relaxation modulus. Such an approach is typical in the context of the inverse problem. 

Here, as a measure of the model (9), equivalently (14), the accuracy of the index $J\left(g_{K}\right)$ of the form (16) is applied. Note, that the first component of $J\left(g_{K}\right)$ given by the right-hand side of (19) depends on the unknown relaxation spectrum; the second term is determined by model parameters $g_{k}$ and the values of the measurable relaxation modulus at the time instant $t_{k}=\alpha k$; and the last component is affected only by the model parameters and the times $t_{k}$. Replacing in (19) the relaxation modulus $G\left(t_{k}\right)=G\left(\alpha k\right)$ by their measurements $\bar{G}\left(t_{k}\right)$, we obtain the following integral-empirical index

(24) $${\bar{J}}_{K}\left(g_{K}\right)=\int_{0}^{\infty }{H^{M}\left(v\right)}^{2}dv-2\sum_{k=1}^{K}g_{k}\bar{G}\left(\alpha k\right)+\sum_{k=1}^{K}\sum_{m=1}^{K}g_{k}g_{m}\varphi_{km}.$$

Let us introduce the vector-matrix notation

(25) $$\Phi_{K}=\left[\begin{array}{ccc} \frac{1}{2} & \cdots & \frac{1}{K+1}\\ \vdots & \ddots & \vdots \\ \frac{1}{K+1} & \cdots & \frac{1}{2K} \end{array}\right],\;{\bar{G}}_{K}=\left[\begin{array}{c} \bar{G}\left(t_{1}\right)\\ \vdots \\ \bar{G}\left(t_{K}\right) \end{array}\right].$$

Note that element (k,m) of the matrix $\Phi_{K}$, i.e., the entry in the k-th row and m-th column of $\Phi_{K}$, is equal to $\alpha \varphi_{km}$ and is dimensionless. Therefore, the algebraic properties of the matrix $\Phi_{K}$ do not depend on the time-scale factor α. Using the above notation and bearing in mind (18), the identification index (24) can be expressed in compact form as follows:

(26) $${\bar{J}}_{K}\left(g_{K}\right)=\int_{0}^{\infty }{H^{M}\left(v\right)}^{2}dv-2{\bar{G}}_{K}^{T}g_{K}+\frac{1}{\alpha }g_{K}^{T}\Phi_{K}g_{K}.$$

Thus, the optimal identification of the relaxation spectrum in the class of models (9), equivalently (14), consists of solving—with respect to the model parameter $g_{K}$—the linear-quadratic problem

(27) $$\underset{g_{K}\in R^{K}}{min}{\bar{J}}_{K}\left(g_{K}\right),$$

the system of normal equations of which is as follows:

(28) $$\Phi_{K}g_{K}=\alpha {\bar{G}}_{K}.$$

The existence and properties of the solution to (28) depend on the properties of square symmetric matrix $\Phi_{K}$ (25) specified by the following result, which is the simple consequence of the independence of the basis functions $h_{k}\left(v\right)$ (7), as proved in Appendix A.1.

**Lemma 1.**  *The matrix* $\Phi_{K}$, *Equation (25), is positive definite for an arbitrary* K≥1.

By the above lemma, the unique solution of the minimization task (27) is as follows:

(29) $$g_{K}=\alpha {\Phi_{K}^{-1}\bar{G}}_{K}.$$

The matrix $\Phi_{K}$, although of full-rank, is extremely ill-conditioned and must be used with care. Namely, the problem of the matrix $\Phi_{K}$ inversion is ill-conditioned, therefore small perturbations in $\Phi_{K}$ may produce large changes in $\Phi_{K}^{-1}$. The spectral condition number ([39] Equation (2.6.3))

(30) $$\kappa \left(\Phi_{K}\right)={\left\|\Phi_{K}^{-1}\right\|}_{2}\cdot {\left\|\Phi_{K}\right\|}_{2},$$

where ${\left\|\cdot \right\|}_{2}$ is the spectral norm of matrices, and equal, in fact, to the ratio of the largest singular value of $\Phi_{K}$ to the smallest, measures the sensitivity of the answer to small perturbation of the data. From the first row values in Table 1, where $\kappa \left(\Phi_{K}\right)$ is given for a few values of K, we see that index $\kappa \left(\Phi_{K}\right)$ exceeds the value of 10^5^ already for K=5, the value of 10^10^ as early as K=8 and tends to infinity with growing K. Thus, positive definite $\Phi_{K}$ is suspected to be very ill-conditioned, even for not very large K≥5, and numerical solution of (28) results in fluctuations of the parameters vector $g_{K}$, which are the greater, the greater is the value of the condition number $\kappa \left(\Phi_{K}\right)$. 

Summarizing, the linear-quadratic identification task (27) is ill-conditioned [40] and when the data are noisy even small changes in ${\bar{G}}_{K}$ would lead to an arbitrarily large artefact in $g_{K}$ given by (29). Therefore, the numerical solution of the finite-dimensional problem (27) is fraught with the same difficulties that the original continuous ill-posed problems of numerical solution of the Fredholm Equations (2) or (5).

### 3.3. Regularization

To deal with the ill-conditioning, we use Tikhonov regularization [41], which is classical and because of its simplicity, probably the most common method for solving ill-posed linear-quadratic problems. For the linear-quadratic task (27), Tikhonov regularization strives in minimizing a modified square functional of the form

(31) $${\bar{J}}_{K}\left(g_{K}\right)+\lambda g_{K}^{T}g_{K},$$

where λ>0 is a regularization parameter. The unit of λ must be $s^{-1}$ to ensure dimensional consistency of the above index. For ${\bar{J}}_{K}\left(g_{K}\right)$ given by (26), the regularized task results in the linear-quadratic optimization task

(32) $$\underset{g_{K}\in R^{K}}{min}\frac{1}{\alpha }g_{K}^{T}\Phi_{K}g_{K}-2{\bar{G}}_{K}^{T}g_{K}+\lambda g_{K}^{T}g_{K};$$

the first summand of (26), being independent on $g_{K}$, does not have to be taken into account here, just as it did not affect the minimization result in the original problem (27). The set of normal equations is now as follows

(33) $$\left(\Phi_{K}+\alpha \lambda I_{K}\right)g_{K}=\alpha {\bar{G}}_{K},$$

where $I_{K}$ is K×K identity matrix. 

The existence and properties of the solution of (33) depend on the properties of the symmetric matrix $\left(\Phi_{K}+\alpha \lambda I_{K}\right)$. Based on Lemma 1, $\left(\Phi_{K}+\alpha \lambda I_{K}\right)$ is non-singular and positive definite for any λ≥0. In successive rows of Table 1, the spectral conditional numbers $\kappa \left(\Phi_{K}+\alpha \lambda I_{K}\right)$ are given for a few values of the dimensionless product αλ, which determines the value of $\kappa \left(\Phi_{K}+\alpha \lambda I_{K}\right)$ for given K. The numerical studies indicate that $\kappa \left(\Phi_{K}+\alpha \lambda I_{K}\right)$ is not greater than the numerically acceptable value 10^5^ [42] for K≤50, whenever the parameters product $\alpha \lambda \geq 1.7\times 10^{-5}$, for K≤100 if $\alpha \lambda \geq 1.89\times 10^{-5}$, and for $\alpha \lambda \geq 2.16\times 10^{-5}$, assuming measurement points K≤500. 

Therefore, the problem (32) is well-posed, that is the solution exists, is unique, and continuously depends on both the matrix $\Phi_{K}$ and the measurements ${\bar{G}}_{K}$. By (33), the optimal regularized vector is given by the following formula:

(34) $${\bar{g}}_{K}\left(\lambda \right)=\alpha {\left(\Phi_{K}+\alpha \lambda I_{K}\right)}^{-1}{\bar{G}}_{K}.$$

Elegant and compact Formula (34) is, however, unsuitable for computational purposes, for which the singular decomposition technique [39] will be used.

### 3.4. Algebraic Background

Let the singular value decomposition (SVD) of the matrix $\Phi_{K}$ (25) take the form [39]

(35) $$\Phi_{K}=U_{K}\Sigma_{K}U_{K}^{T},$$

where the diagonal K×K matrix

(36) $$\Sigma_{K}=diag\left(\sigma_{1},\ldots ,\sigma_{K}\right),$$

is composed of the non-zero singular values $\sigma_{1}\geq \ldots \geq \sigma_{k}\geq \ldots \geq \sigma_{K}$ of the matrix $\Phi_{K}$; matrix $U_{K}\in R^{K,K}$ is orthogonal. The SVD (35) is uniquely determined. The singular values $\sigma_{k}$ of $\Phi_{K}$ do not depend on the time-scale factor α. Therefore, for given K, the SVD must be computed only once even if the sampling instants $t_{k}=\alpha k$ dependent on the parameter α are changed in the experiment. Taking advantage of the diagonal structure of $\Sigma_{K}$ and orthogonality of the matrix $U_{K}$, we have:

(37) $${\left(\Phi_{K}+\alpha \lambda I_{K}\right)}^{-1}={U_{K}\left(\Sigma_{K}+\alpha \lambda I_{K}\right)}^{-1}U_{K}^{T}=U_{K}\Omega_{K}{U_{K}^{T}}^{T},$$

where diagonal K×K matrix

(38) $${\Omega_{K}=\left(\Sigma_{K}+\alpha \lambda I_{K}\right)}^{-1}=diag\left(\frac{1}{\sigma_{1}+\alpha \lambda },\ldots ,\frac{1}{\sigma_{K}+\alpha \lambda }\right).$$

In view of (37), (34), and (38), the optimal regularized vector ${\bar{g}}_{K}\left(\lambda \right)$ is expressed as follows:

(39) $${\bar{g}}_{K}\left(\lambda \right)=\alpha {U_{K}\left(\Sigma_{K}+\alpha \lambda I_{K}\right)}^{-1}Y_{K}=\alpha U_{K}\Omega_{K}Y_{K},$$

where the K dimensional vector

(40) $$Y_{K}=U_{K}^{T}{\bar{G}}_{K}.$$

According to (9), (10), and (14), the resulting best relaxation spectra models are as follows

(41) $${\bar{H}}_{K}^{M}\left(v\right)=\sum_{k=1}^{K}{\bar{g}}_{k}\left(\lambda \right)h_{k}\left(v\right),$$

(42) $${\bar{H}}_{K}\left(v\right)=\sum_{k=1}^{K}{\bar{g}}_{k}\left(\lambda \right)h_{k}\left(v\right)v,$$

and

(43) $${\bar{H}}_{K}\left(\tau \right)=\sum_{k=1}^{K}{\bar{g}}_{k}\left(\lambda \right)h_{k}\left(\tau \right),$$

where ${\bar{g}}_{k}\left(\lambda \right)$ are elements of the vector ${\bar{g}}_{K}\left(\lambda \right)$ (34), or equivalent (39). 

### 3.5. Analysis

The model smoothing and its accuracy in the case of noisy measurements of the relaxation modulus will be now analyzed. Contrary to the previous papers [26,27,28]—in which the model quality index refers to the relaxation modulus but not directly to the unknown relaxation spectrum—here we can estimate the spectra errors ${\left\|H^{M}\left(v\right)-{\bar{H}}_{K}^{M}\left(v\right)\right\|}_{2}$, ${\left\|{\bar{H}}_{K}\left(v\right)-{\tilde{H}}_{K}\left(v\right)\right\|}_{2}$, and ${\left\|H\left(\tau \right)-{\bar{H}}_{K}\left(\tau \right)\right\|}_{2}$ directly, where ${\left\|\cdot \right\|}_{2}$ denotes the square norm in the space $L^{2}\left(0,\right.\left.\infty \right)$.

#### 3.5.1. Model Smoothness 

The purpose of the regularization applied in (31) relies on stabilization of the vector of model parameters ${\bar{g}}_{K}\left(\lambda \right)$ (34). The norms ${\left\|{\bar{H}}_{K}^{M}\left(v\right)\right\|}_{2}$, ${\left\|{\bar{H}}_{K}\left(v\right)\right\|}_{2}$, and ${\left\|{\bar{H}}_{K}\left(\tau \right)\right\|}_{2}$ are natural measures of the spectra models’ (41)–(43) smoothness. In Appendix A.2, the following result is derived.

**Proposition 1.**  *Let the time-scale factor α>0 and the regularization parameter λ>0. Then, for the optimal relaxation spectra models ${\bar{H}}_{K}^{M}\left(v\right)$ (41), ${\bar{H}}_{K}\left(v\right)$ (42), and ${\bar{H}}_{K}\left(\tau \right)$ (43) we have the following*

(44) $${\frac{1}{\alpha }\sigma_{K}{\bar{g}}_{K}^{T}\left(\lambda \right){\bar{g}}_{K}\left(\lambda \right)\leq \left\|{\bar{H}}_{K}^{M}\left(v\right)\right\|}_{2}^{2}={\left\|{\bar{H}}_{K}\left(\tau \right)\right\|}_{2}^{2}=\frac{1}{\alpha }{\bar{g}}_{K}^{T}\left(\lambda \right)\Phi_{K}{\bar{g}}_{K}\left(\lambda \right)\leq \;\frac{1}{\alpha }\sigma_{1}{\bar{g}}_{K}^{T}\left(\lambda \right){\bar{g}}_{K}\left(\lambda \right),$$

*where the vector ${\bar{g}}_{K}\left(\lambda \right)$ is given by (39) and*

(45) $$\frac{2}{\alpha^{3}}\varsigma_{K}{\bar{g}}_{K}^{T}\left(\lambda \right){\bar{g}}_{K}\left(\lambda \right)\leq {\left\|{\bar{H}}_{K}\left(v\right)\right\|}_{2}^{2}=\frac{2}{\alpha^{3}}{\bar{g}}_{K}^{T}\left(\lambda \right)\Theta_{K}{\bar{g}}_{K}\left(\lambda \right)\leq \frac{2}{\alpha^{3}}\varsigma_{1}{\bar{g}}_{K}^{T}\left(\lambda \right){\bar{g}}_{K}\left(\lambda \right),$$

*where $\sigma_{K}$ and $\sigma_{1}$ are the minimal and maximal singular values of the matrix $\Phi_{K}$ (25), while $\varsigma_{K}$ and $\varsigma_{1}$ are the minimal and maximal singular values of the positive definite matrix*

(46) $$\Theta_{K}=\left[\begin{array}{ccc} \frac{1}{2^{3}} & \cdots & \frac{1}{{\left(K+1\right)}^{3}}\\ \vdots & \ddots & \vdots \\ \frac{1}{{\left(K+1\right)}^{3}} & \cdots & \frac{1}{{\left(2K\right)}^{3}} \end{array}\right].$$

The values of square roots of the smallest and largest singular values $\sigma_{K}$, $\sigma_{1}$, $\varsigma_{K}$, and $\varsigma_{1}$ for some model summands K are summarized in Table 2. Due to the ill-conditioning of matrices $\Phi_{K}$ and $\Theta_{K}$, the lower bounds in (44) and (45) are not too useful. Since $\sqrt{\sigma_{1}}$ and $\sqrt{\varsigma_{1}}$ grows with K, from the analysis of Table 2 data and the right inequalities in (44) and (45), the next result follows immediately.

**Proposition 2.**  *Let the time-scale factor α>0 and the regularization parameter λ>0. If the number of relaxation modulus measurements $K\leq 10^{4}$, then the optimal relaxation spectra models ${\bar{H}}_{K}^{M}\left(v\right)$ (41), ${\bar{H}}_{K}\left(v\right)$ (42), and ${\bar{H}}_{K}\left(\tau \right)$ (43) are such that*

(47) $${\left\|{\bar{H}}_{K}^{M}\left(v\right)\right\|}_{2}={\left\|{\bar{H}}_{K}\left(\tau \right)\right\|}_{2}\leq \frac{1.5747}{\sqrt{\alpha }}{\left\|{\bar{g}}_{K}\left(\lambda \right)\right\|}_{2},$$

(48) $${\left\|{\bar{H}}_{K}\left(v\right)\right\|}_{2}\leq \frac{0.5285}{\alpha \sqrt{\alpha }}{\left\|{\bar{g}}_{K}\left(\lambda \right)\right\|}_{2},$$

*where the vector ${\bar{g}}_{K}\left(\lambda \right)$ (39) and ${\left\|\cdot \right\|}_{2}$ denotes here the square norm in Euclidean space* $R^{K}$.

Since, by the right equality in (39) and the orthogonality of $U_{K}$, we obtain:

$${\bar{g}}_{K}^{T}\left(\lambda \right){\bar{g}}_{K}\left(\lambda \right)=\alpha^{2}Y_{K}^{T}{\Omega_{K}U_{K}^{T}U}_{K}\Omega_{K}Y_{K}^{T}=\alpha^{2}Y_{K}^{T}\Omega_{K}^{2}Y_{K}^{T},$$

bearing in mind the diagonal structure of $\Omega_{K}$ (38) we have the formula:

(49) $${{\left\|{\bar{g}}_{K}\left(\lambda \right)\right\|}_{2}^{2}=\bar{g}}_{K}^{T}\left(\lambda \right){\bar{g}}_{K}\left(\lambda \right)=\alpha^{2}\sum_{k=1}^{K}\;\frac{y_{k}^{2}}{{\left(\sigma_{k}+\alpha \lambda \right)}^{2}},$$

where $y_{k}$ are the elements of the vector $Y_{K}$ (40), which directly illustrates the mechanism of the regularization. The following rule holds: the greater the regularization parameter λ is, the more highly bounded the fluctuations of the vector ${\bar{g}}_{K}\left(\lambda \right)$ are.

Summarizing, the smoothness of the optimal vector ${\bar{g}}_{K}\left(\lambda \right)$ of the model’s parameters guarantees that the fluctuations of the resulting relaxation spectra models ${\bar{H}}_{K}^{M}\left(v\right)$, ${\bar{H}}_{K}\left(\tau \right)$, and ${\bar{H}}_{K}\left(v\right)$ are also bounded. Both time-scale factor α and the regularization parameter λ affect the smoothness of the spectrum models. However, it should be remembered that the inequalities (47) and (48) give only the upper bounds of the respective norms.

#### 3.5.2. Noise Robustness and Convergence

The model of the modified spectrum that we would obtain for the same time-scale factor α and regularization parameter λ on the basis of ideal (noise-free) relaxation modulus measurements:

(50) $${\tilde{H}}_{K}^{M}\left(v\right)=\sum_{k=1}^{K}{\tilde{g}}_{k}\left(\lambda \right)h_{k}\left(v\right),$$

where ${\tilde{g}}_{K}\left(\lambda \right)$ is the vector model parameters given by the following (compare (39) and (40)):

(51) $${\tilde{g}}_{K}\left(\lambda \right)=\alpha U_{K}\Omega_{K}U_{K}^{T}G_{K}$$

for the noise-free relaxation modulus $G_{K}={\left[\begin{array}{ccc} G\left(t_{1}\right) & \cdots & G\left(t_{K}\right) \end{array}\right]}^{T}$, which will be considered as a reference point for the model ${\bar{H}}_{K}^{M}\left(v\right)$ (41). The respective noise-free optimal regularized models of the relaxation frequency and time spectra are as follows

(52) $${\tilde{H}}_{K}\left(v\right)=\sum_{k=1}^{K}{\tilde{g}}_{k}\left(\lambda \right)h_{k}\left(v\right)v,$$

and

(53) $${\tilde{H}}_{K}\left(\tau \right)=\sum_{k=1}^{K}{\tilde{g}}_{k}\left(\lambda \right)h_{k}\left(\tau \right).$$

In Appendix A.3, the following estimations are derived.

**Proposition 3.**  *For an arbitrary time-scale factor α and arbitrary regularization parameter λ, the errors between the relaxation spectra models ${\bar{H}}_{K}^{M}\left(v\right)$ (41), ${\bar{H}}_{K}\left(v\right)$ (42), and ${\bar{H}}_{K}\left(\tau \right)$ (43) and related noise-free models ${\tilde{H}}_{K}^{M}\left(v\right)$ (50), ${\tilde{H}}_{K}\left(v\right)$ (52), and ${\tilde{H}}_{K}\left(\tau \right)$ (53) are estimated by the following inequalities:*

(54) $${\left\|{\bar{H}}_{K}^{M}\left(v\right)-{\tilde{H}}_{K}^{M}\left(v\right)\right\|}_{2}={\left\|{{\bar{H}}_{K}\left(\tau \right)-\tilde{H}}_{K}\left(\tau \right)\right\|}_{2}\leq \sqrt{\alpha }\gamma {\left\|z_{N}\right\|}_{2},$$

 *where parameter*

(55) $$\gamma =\underset{1\leq k\leq K}{\max}\;\frac{\sqrt{\sigma_{k}}}{\sigma_{k}+\alpha \lambda },$$

 *and*

(56) $${\left\|{\bar{H}}_{K}\left(v\right)-{\tilde{H}}_{K}\left(v\right)\right\|}_{2}\leq \frac{\sqrt{2\;\varsigma_{1}}}{\sqrt{\alpha }\left(\sigma_{K}+\alpha \lambda \right)}{\left\|z_{N}\right\|}_{2},$$

*where $\varsigma_{1}$ is the maximal singular value of $\Theta_{K}$ (46); $z_{N}={\left[\begin{array}{ccc} z\left(t_{1}\right) & \cdots & z\left(t_{N}\right) \end{array}\right]}^{T}$ is the vector of measurement noises.*

According to inequalities (54) and (56), the accuracy of the noise-free optimal spectra approximation depends on the measurement noises, the regularization parameter, the time-scale factor, and on the singular values of the matrices $\Phi_{K}$ (25) and $\Theta_{K}$ (46), this being dependent on the number of measurements. By (54) and (56), having in mind the continuity of all the spectra considered here, we conclude that the spectra ${\bar{H}}_{K}^{M}\left(v\right)$ (41), ${\bar{H}}_{K}\left(v\right)$ (42), and ${\bar{H}}_{K}\left(\tau \right)$ (43) tend to their noise-free counterparts for each v>0 and τ>0 linearly with respect to the norm ${\left\|z_{N}\right\|}_{2}$, as ${\left\|z_{N}\right\|}_{2}\to 0$, and the faster the larger the regularization parameter λ. 

#### 3.5.3. Error of the Relaxation Modulus Model

The approximation of the material spectrum $H\left(v\right)$ by series of functions $H_{K}^{M}\left(v\right)$ (9) results in the relaxation modulus $G\left(t\right)$ approximation by the series $G_{K}\left(t\right)$ (11) of basis functions $\phi_{k}\left(t\right)$ (12). Therefore, the relaxation modulus model corresponding to the relaxation spectra models (41)–(43) is described by the following equation:

(57) $${\bar{G}}_{K}\left(t\right)=\sum_{k=1}^{K}{\bar{g}}_{k}\left(\lambda \right)\phi_{k}\left(t\right).$$

The mean square error of the relaxation modulus model is as follows

(58) $$Q_{K}\left({\bar{g}}_{K}\left(\lambda \right)\right)=\frac{1}{K}\sum_{k=1}^{K}{\left[\bar{G}\left(t_{k}\right)-{\bar{G}}_{K}\left(t_{k}\right)\right]}^{2}.$$

In Appendix A.4, the following result is derived.

**Proposition 4.**  *For an arbitrary time-scale factor α and arbitrary regularization parameter λ, the square error of the relaxation modulus model ${\bar{G}}_{K}\left(t\right)$ (57) defined by (58) for the optimal model parameter ${\bar{g}}_{K}\left(\lambda \right)$ (39) is given by the following formula:*

(59) $$Q_{K}\left({\bar{g}}_{K}\left(\lambda \right)\right)=\frac{1}{K}{\left[{\bar{G}}_{K}-\frac{1}{\alpha }\Phi_{K}{\bar{g}}_{K}\left(\lambda \right)\right]}^{T}\left[{\bar{G}}_{K}-\frac{1}{\alpha }\Phi_{K}{\bar{g}}_{K}\left(\lambda \right)\right]=\sum_{k=1}^{K}\frac{y_{k}^{2}}{{\left(\frac{\sigma_{k}}{\alpha \lambda }+1\right)}^{2}},$$

*where $\sigma_{k}$, k=1,…,K, are the singular values of the matrix; $\Phi_{K}$ (25) and $y_{k}$ are the elements of the vector $Y_{K}$ (40).*

The equality (59) yields that the accuracy of the relaxation modulus approximation depends on the following: the real relaxation modulus $G_{K}$ and measurement noises $z_{K}$ affecting the value of $Y_{K}=U_{K}^{T}G_{K}+U_{K}^{T}z_{K}$; the scaling factor α; the regularization parameter λ; and singular values of the matrix $\Phi_{K}$ (25). These, in turn, depend on the number of measurements. Note also that only the product αλ, not α and λ independently, affects the index $Q_{K}\left({\bar{g}}_{K}\left(\lambda \right)\right)$. Since the first derivative

$$\frac{\partial Q_{K}\left({\bar{g}}_{K}\left(\lambda \right)\right)}{\partial \left(\alpha \lambda \right)}=2\sum_{k=1}^{K}\frac{y_{k}^{2}\sigma_{k}\alpha \lambda }{{\left(\sigma_{k}+\alpha \lambda \right)}^{3}},$$

is positive for any αλ>0, the error of the relaxation modulus model grows with increasing regularization parameter λ, slow for very small and very large αλ. 

### 3.6. Identification Algorithm

Allowing the above, the calculation of the relaxation spectra models involves the following steps.

1. For the studied material, perform the preliminary experiment (stress relaxation test [1,33,38]) and record the measurements $\bar{G}\left(t_{i}\right)$, i=1,…,N, of the relaxation modulus for pre-selected time instants (e.g., sampled with the constant period in the time interval $\left[0,T\right]$, T<∞);
2. Choose the time-scaling factor α and the number K of model components comparing, for different values of α, a few functions from the sequence $\left\{\phi_{k}\left(t\right)\right\}$ given by (12), and creating relaxation modulus model $G_{K}\left(t\right)$ (11) with the experiment results $\left\{\bar{G}\left(t_{i}\right)\right\}$;
3. Perform the experiment and record the measurements $\bar{G}\left(t_{k}\right)$ of the relaxation modulus at times $t_{k}=\alpha \cdot k$, k=1,…,K;
4. Compute the matrix $\Phi_{K}$ (25), and next, determine the SVD (35) with the singular values $\sigma_{1},\ldots ,\sigma_{k},\ldots \sigma_{K}$ of $\Phi_{K}$;
5. Select the regularization parameter λ such that for assumed α and K the spectral condition number is such that
  (60) $$\kappa \left(\Phi_{K}+\alpha \lambda I_{K,K}\right)=\frac{\sigma_{1}+\alpha \lambda }{\sigma_{K}+\alpha \lambda }\leq 10^{5};$$
6. For chosen λ, compute the regularized solution ${\bar{g}}_{K}\left(\lambda \right)$ according to (39);
7. Determine the modified spectrum of relaxation frequencies ${\bar{H}}_{K}^{M}\left(v\right)$ according to (41);
8. Determined the spectra of relaxation time ${\bar{H}}_{K}\left(\tau \right)$ and frequency ${\bar{H}}_{K}\left(v\right)$ according to (43) and (42), respectively, as the linear combinations of the respective basis functions.

The matrix $\Phi_{K}$ (25) and, in particular, the singular values $\sigma_{k}$ of $\Phi_{K}$ depend only on the number of measurements and do not depend even on the time-scale factor α and on the sampling points $t_{k}$. Therefore, for the fixed K matrix $\Phi_{K}$ and the SVD of $\Phi_{K}$—being the most space- and time-consuming task of the scheme of computational complexity $O\left(NK^{2}\right)$ [39]—these must be determined only once when the identification scheme is applied for successive samples of the same material (step 4). The SVD is accessible in the form of optimized numerical procedures in most computational packets. 

The condition (60) from step 5 means that the condition number does not exceed the numerically acceptable value 10^5^ [42]; data from Table 1 may be useful here. 

### 3.7. Simulational Studies

**Figure 5:**
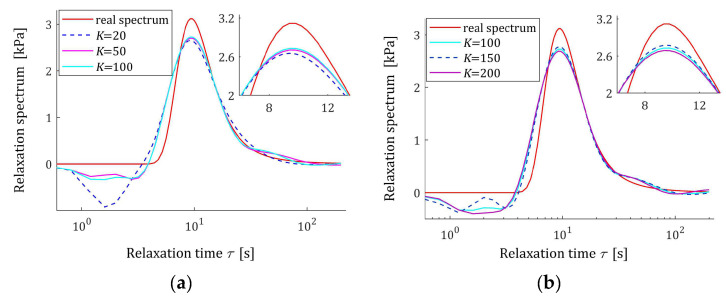
Uni-mode Gauss-like time relaxation spectrum $H\left(\tau \right)$ (65) (solid red line) and the corresponding models ${\bar{H}}_{K}\left(\tau \right)$ (43) for K measurements of the relaxation modulus corrupted by additive independent noises uniformly distributed over the interval $\left[-10,\;10\right]\;Pa$: (**a**) K=20, 50, 100; (**b**) K=100, 150, 200.

**Figure 6:**
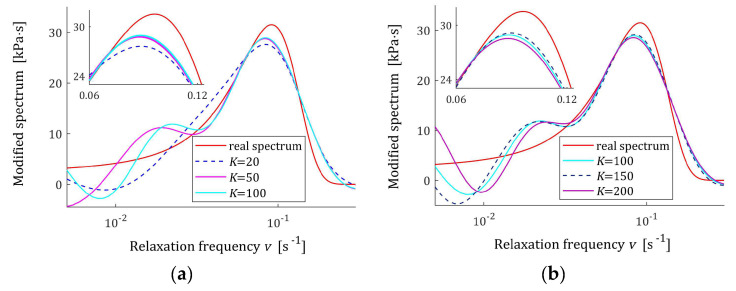
Modified uni-mode Gauss-like time relaxation spectrum $H^{M}\left(v\right)$ (69) (solid red line) and the corresponding models ${\bar{H}}_{K}^{M}\left(v\right)$ (41) for K measurements of the relaxation modulus corrupted by additive independent noises uniformly distributed over the interval $\left[-10,\;10\right]\;Pa$: (**a**) K=20, 50, 100; (**b**) K=100, 150, 200.

**Figure 7:**
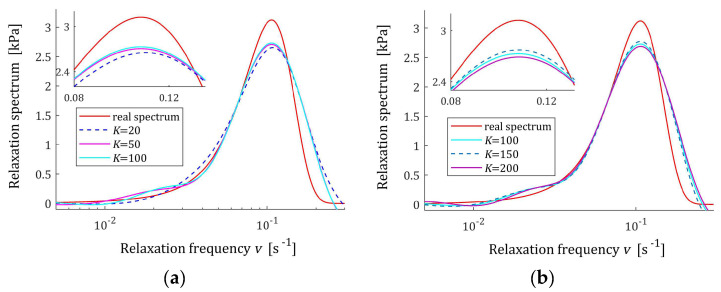
Uni-mode Gauss-like time relaxation frequency spectrum $H\left(v\right)$ (68) (solid red line) and the corresponding models ${\bar{H}}_{K}\left(v\right)$ (42) for K measurements of the relaxation modulus corrupted by additive independent noises uniformly distributed over the interval $\left[-10,\;10\right]\;Pa$: (**a**) K=20, 50, 100; (**b**) K=100, 150, 200.

**Figure 8:**
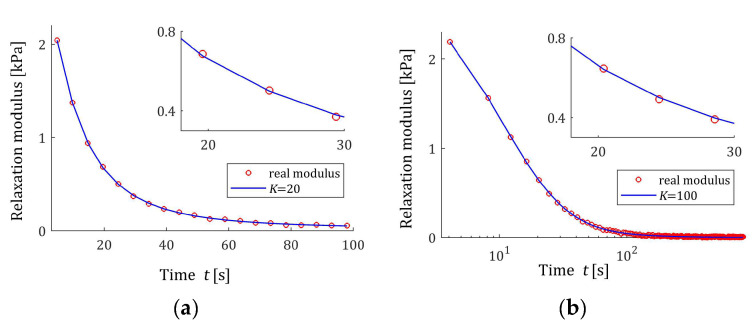
The measurements $\bar{G}\left(t_{k}\right)$ of uni-mode Gauss-like time relaxation modulus $G\left(t\right)$ (66) corrupted by additive independent noises uniformly distributed over the interval $\left[-10,\;10\right]\;Pa$ (red points) and the corresponding relaxation modulus models ${\bar{G}}_{K}\left(t\right)$ (57) for K measurements of the relaxation modulus: (**a**) K=20; (**b**) K=200.

**Figure 9:**
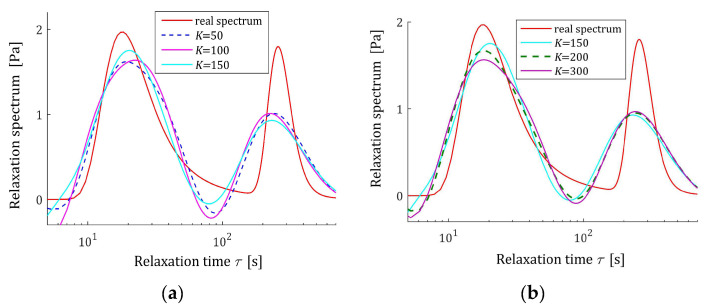
Double-mode Gauss-like time relaxation spectrum $H\left(\tau \right)$ (70) (solid red line) and the corresponding models ${\bar{H}}_{K}\left(\tau \right)$ (43) for K measurements of the relaxation modulus corrupted by additive independent noises uniformly distributed over the interval $\left[-0.005,\;0.005\right]\;Pa$: (**a**) K=50, 100, 150; (**b**) K=150, 200, 300.

**Figure 10:**
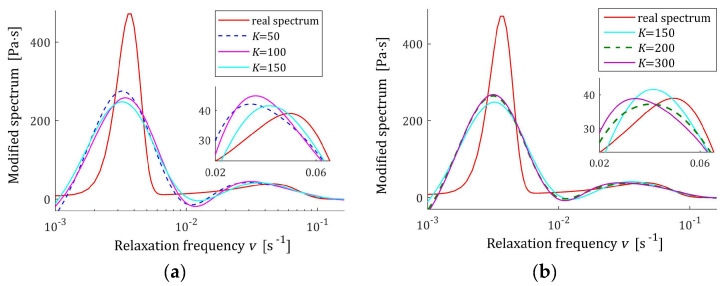
Modified double-mode Gauss-like time relaxation spectrum $H^{M}\left(v\right)$ related to $H\left(v\right)$ (71) (solid red line) and the corresponding models ${\bar{H}}_{K}^{M}\left(v\right)$ (41) for K measurements of the relaxation modulus corrupted by additive independent noises uniformly distributed over the interval $\left[-0.005,\;0.005\right]\;Pa$: (**a**) K=50, 100, 150; (**b**) K=150, 200, 300.

**Figure 11:**
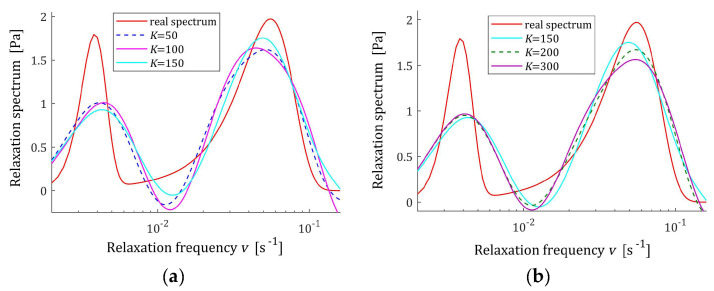
Double-mode Gauss-like time relaxation frequency spectrum $H\left(v\right)$ (71) (solid red line) and the corresponding models ${\bar{H}}_{K}\left(v\right)$ (42) for K measurements of the relaxation modulus corrupted by additive independent noises uniformly distributed over the interval $\left[-0.005,\;0.005\right]\;Pa$: (**a**) K=50, 100, 150; (**b**) K=150, 200, 300.

This section presents the results of the approach with proposed numerical studies for three simulated materials whose viscoelastic properties are described by the Gauss-like and Kohlrausch–Williams–Watts (KWW) models. Gauss distributions of the relaxation spectra are examined while developing new identification methods; the best examples are as follows: ([5] (Figure 2)), ([14] (Figures 9, 11 and 17)) and ([43] (Figures 2, 3, 6–11 and 14)). The Gaussian-like distributions of the relaxation spectra were used to describe the viscoelastic properties of a lot of real materials, mainly polymers, for example, poly(methyl methacrylate) [44], polyacrylamide gels ([45] (Figure A4)), polyethylene [46] and carboxymethylcellulose (CMC) [47]. Gaussian nature has also the spectra of many biopolymers, e.g., fresh egg white-hydrocolloids [47], cold gel-like emulsions stabilized with bovine gelatin [48], xanthan gum water solution [47], some (potato, corn, wheat, and banana) native starch gels [49], and wood [24,50]. Recently, Gaussian-type relaxation spectra have also been determined for the modified asphalt binder blends ([51] (Figures 3a,c and 5a,c,e)).

The KWW model of the stretched exponential relaxation has been found by many researchers to be more appropriate than standard exponentials to describe viscoelastic processes of many materials, for example, polymer melts [52], the segmental dynamics and the glass transition behavior of poly(2-vinylpyridine) [53], relaxation of bone and bone collagen [54], and alginate films while considering glycerol concentration [55]. The KWW model, initially introduced to describe the viscoelastic relaxation processes [56,57], has also been used to model other relaxation processes occurring in materials, for example, enthalpy relaxation in Cu_46_Zr_45_Al_7_Y_2_ and Zr_55_Cu_30_Ni_5_Al_10_ bulk metallic glasses [58], isothermal enthalpy relaxation and density relaxation investigated for bulk Pd_42.5_Cu_30_Ni_7.5_P_20_ and Pd_40_Ni_40_P_20_ metallic glasses [59], and structural relaxation of a Hf-microalloyed Co-based glassy alloy [60]. 

Applying the proposed identification algorithm, the best spectra models ${\bar{H}}_{K}^{M}\left(v\right)$ (41), ${\bar{H}}_{K}\left(v\right)$ (42), and ${\bar{H}}_{K}\left(\tau \right)$ (43) were determined for a few numbers of measurements. The smoothness of the models is estimated by their integral square norms described in Proposition 1. For the spectra ${\bar{H}}_{K}^{M}\left(v\right)$ and ${\bar{H}}_{K}\left(\tau \right)$, these norms are equal and uniquely characterized by the middle equality in (44), which yields the following:

(61) $${\left\|{\bar{H}}_{K}^{M}\left(v\right)\right\|}_{2}={\left\|{\bar{H}}_{K}\left(\tau \right)\right\|}_{2}=\frac{1}{\sqrt{\alpha }}\sqrt{{\bar{g}}_{K}^{T}\left(\lambda \right)\Phi_{K}{\bar{g}}_{K}\left(\lambda \right)}.$$

By (23), for arbitrary relaxation spectra $H\left(\tau \right)$ and $H^{M}\left(v\right)$ of the real material, the analogous equality of the norms holds, i.e., ${\left\|H^{M}\left(v\right)\right\|}_{2}={\left\|H\left(\tau \right)\right\|}_{2}$. The formula describing the square norm of ${\bar{H}}_{K}\left(v\right)$ (42) directly results from the equality in (45), from which the following can be obtained:

(62) $${\left\|{\bar{H}}_{K}\left(v\right)\right\|}_{2}=\frac{\sqrt{2}}{\alpha \sqrt{\alpha }}\sqrt{{\bar{g}}_{K}^{T}\left(\lambda \right)\Theta_{K}{\bar{g}}_{K}\left(\lambda \right)},$$

with the matrix $\Theta_{K}$ defined by (46).

The errors of the relaxation modulus models are estimated using index $Q_{K}\left({\bar{g}}_{K}\left(\lambda \right)\right)$ (58) and expressed for the optimal models ${\bar{H}}_{K}^{M}\left(v\right)$ (41), ${\bar{H}}_{K}\left(v\right)$ (42), and ${\bar{H}}_{K}\left(\tau \right)$ (43) by Formula (59) from Proposition 4. 

The errors of the relaxation spectra models are measured directly by the integral $J\left(g_{K}\right)$, introduced by Equation (16) for model $H_{K}^{M}\left(v\right)$ and by Equation (20) for the relaxation time spectrum model. By (19), having in mind the notation (25), this index for the optimal model ${\bar{H}}_{K}^{M}\left(v\right)$ (41) can be expressed as follows (compare to (26)):

(63) $$J\left({\bar{g}}_{K}\left(\lambda \right)\right)=\int_{0}^{\infty }{H^{M}\left(v\right)}^{2}dv-2G_{K}^{T}{\bar{g}}_{K}\left(\lambda \right)+\frac{1}{\alpha }{\bar{g}}_{K}^{T}\left(\lambda \right)\Phi_{K}{\bar{g}}_{K}\left(\lambda \right),$$

where $G_{K}$ is the vector of noise-free values of relaxation modulus defined below Equation (51). The error $J\left({\bar{g}}_{K}\left(\lambda \right)\right)$ for the model ${\bar{H}}_{K}\left(\tau \right)$ (43) is obviously identical due to the identity of indices (16) and (20) proved above. 

The error $J\left({\bar{g}}_{K}\left(\lambda \right)\right)$ related to square of the norm of real spectra ${\left\|H^{M}\left(v\right)\right\|}_{2}^{2}={\left\|H\left(\tau \right)\right\|}_{2}^{2}$ is measured by the relative index

(64) $$J_{rel}\left({\bar{g}}_{K}\left(\lambda \right)\right)=\frac{J\left({\bar{g}}_{K}\left(\lambda \right)\right)}{{\left\|H\left(\tau \right)\right\|}_{2}^{2}}.$$

The “real” materials and the optimal models were simulated in Matlab R2023b, The Mathworks, Inc., Natick, MA, USA. For the singular value decomposition procedure, *svd* was applied. 

### 3.8. Identification of Uni-Mode Gauss-like Spectrum

Consider material whose rheological properties are characterized by the uni-modal Gauss-like distribution [10,61]:

(65) $$H\left(\tau \right)=\vartheta e^{-{\left(\frac{1}{\tau }-m\right)}^{2}/q}/\tau ,$$

where the parameters are as follows [10,27,61]: ϑ=31.52 kPa·s, $m=0.0912\;s^{-1}$, and $q=3.25\times 10^{-3}\;s^{-2}$. The relaxation modulus is displayed below [10]:

(66) $$G\left(t\right)=\frac{\sqrt{\pi q}}{2}\vartheta \;e^{\frac{1}{4}t^{2}q-mt}erfc\left(\frac{\frac{1}{2}tq-m}{\sqrt{q}}\right),$$

where the complementary error function $erfc\left(x\right)$ is defined as follows ([62] Equation (8.250.4)):

(67) $$erfc\left(x\right)=\frac{2}{\sqrt{\pi \;}\;}\int_{x}^{\infty }e^{{-z}^{2}}dz.$$

By the first equality in (3), the following spectrum of relaxation frequencies corresponds to (65)

(68) $$H\left(v\right)=\vartheta ve^{-{\left(v-m\right)}^{2}/q},$$

whence, in view of (4), the modified spectrum is described as outlined below:

(69) $$H^{M}\left(v\right)=\vartheta e^{-{\left(v-m\right)}^{2}/q}.$$

In Appendix A.5, the analytical Formulas (A10) and (A13) are derived describing the norms of the spectra $H\left(\tau \right)$ (65), $H\left(v\right)$ (68), and $H^{M}\left(v\right)$ (69). These norms are as follows: ${{\left\|H^{M}\left(v\right)\right\|}_{2}=\left\|H\left(\tau \right)\right\|}_{2}=8.422432kPa\cdot s^{1/2}$ and ${\left\|H\left(v\right)\right\|}_{2}=0.805043kPa\cdot s^{-1/2}$. 

The preliminary relaxation test experiment was performed (step 1) and the measurements of the relaxation modulus $G\left(t\right)$ (66) were recorded for 200 s, selected following [10,27,61]. Then, the time scale factors α have been selected by comparison of the courses of experiment results $\left\{\bar{G}\left(t_{i}\right)\right\}$ and basis functions $\phi_{k}\left(t\right)$ (12) for a few k. Next, to simulate the experiment, K sampling instants $t_{k}=\alpha k$ were generated with the constant period α for K=20,50,100,150,200 measurements. Additive measurement noises $z\left(t_{k}\right)$ were selected independently by random choice with uniform distribution on the interval $\left[-10,\;10\right]\;Pa$, i.e., double stronger than noises assumed in the previous papers [10,61]. The measurements $\bar{G}\left(t_{k}\right)$ were recorded. For successive K, the matrix $\Phi_{K}$ (25) and SVD (35) were determined. Next, the regularization parameters λ were selected according to the spectral condition number rule (60); their values are given in Table 3. The optimal model parameters ${\bar{g}}_{K}\left(\lambda \right)$ (39) and the models ${\bar{H}}_{K}^{M}\left(v\right)$ (41), ${\bar{H}}_{K}\left(v\right)$ (42), and ${\bar{H}}_{K}\left(\tau \right)$ (43) were determined. The best models are depicted in Figure 5, Figure 6 and Figure 7 together with the real spectra (65), (68), and (69) marked by red lines. Small subfigures show fitting near the maximum of the real spectrum. The respective relaxation modulus models ${\bar{G}}_{K}\left(t\right)$ (57) are plotted in Figure 8 for K=20 and 200, where the measurements $\bar{G}\left(t_{k}\right)$ of the real modulus $G\left(t\right)$ (66) are also marked; the small subfigures confirm the excellent model fit. For K=200, logarithmic time scale is used. In Table 3, the norms ${\left\|{\bar{H}}_{K}^{M}\left(v\right)\right\|}_{2}={\left\|{\bar{H}}_{K}\left(\tau \right)\right\|}_{2}$ (61), ${\left\|{\bar{H}}_{K}\left(v\right)\right\|}_{2}$ (62), and the norms ${\left\|{\bar{g}}_{K}\left(\lambda \right)\right\|}_{2}$—expressed in (49)—of the optimal model parameters are given. In addition, the integral square approximation index $J\left({\bar{g}}_{K}\left(\lambda \right)\right)$ (63) together with the relative index $J_{rel}\left({\bar{g}}_{K}\left(\lambda \right)\right)$ (64) and the mean square approximation index $Q_{K}\left({\bar{g}}_{K}\left(\lambda \right)\right)$ (58) are provided in Table 3. 

The relative spectrum approximation index $J_{rel}\left({\bar{g}}_{K}\left(\lambda \right)\right)$ (64) does not exceed 5%; additionally, the values of the relaxation modulus approximation index $Q_{K}\left({\bar{g}}_{K}\left(\lambda \right)\right)$ (58) indicate the excellent model fit; models ${\bar{G}}_{K}\left(t\right)$ (57) practically coincide with the measurement points $\bar{G}\left(t_{k}\right)$, see Figure 8. An inspection of Figure 5, Figure 6 and Figure 7 shows that for the number of K≥50 measurements, satisfactory approximation of the relaxation spectra was obtained while maintaining the consistency of the maxima of real spectra and their models. 

### 3.9. Identification of Double-Mode Gauss-like Spectrum

Consider now the viscoelastic material of the relaxation spectrum described by the double-mode Gauss-like distribution considered in [10,27,28,46]:

(70) $$H\left(\tau \right)=\left[\vartheta_{1}e^{-{\left(\frac{1}{\tau }-m_{1}\right)}^{2}/q_{1}}+\vartheta_{2}e^{-{\left(\frac{1}{\tau }-m_{2}\right)}^{2}/q_{2}}\right]/\tau ,$$

where the parameters are as follows [27,28]: $\vartheta_{1}=467\;Pa\cdot s$, $m_{1}=0.0037\;s^{-1}$, $q_{1}=1.124261\times 10^{-6}\;s^{-2}$, $\vartheta_{2}=39\;Pa\cdot s$, $m_{2}=0.045\;s^{-1}$, and $q_{2}=1.173\times 10^{-3}\;s^{-2}$. Therefore, the corresponding spectrum of relaxation frequencies is as follows

(71) $$H\left(v\right)=\vartheta_{1}ve^{-{\left(v-m_{1}\right)}^{2}/q_{1}}+\vartheta_{2}ve^{-{\left(v-m_{2}\right)}^{2}/q_{2}},$$

and, in view of (4), the modified spectrum is described by $H^{M}\left(v\right)=H\left(v\right)/v$. By (66), the related real relaxation modulus is outlined below:

(72) $$G\left(t\right)=\frac{\sqrt{\pi }}{2}\left[\vartheta_{1}\sqrt{q_{1}}\;e^{\frac{1}{4}t^{2}q_{1}-m_{1}t}erfc\left(\frac{\frac{1}{2}tq_{1}-m_{1}}{\sqrt{q_{1}}}\right)+\vartheta_{2}\sqrt{q_{2}}\;e^{\frac{1}{4}t^{2}q_{2}-m_{2}t}erfc\left(\frac{\frac{1}{2}tq_{2}-m_{2}}{\sqrt{q_{2}}}\right)\right].$$

In Appendix A.6, the analytical Formulas (A17) and (A19) are derived to describe the square norms of the “real” spectra $H\left(v\right)$ (71), $H\left(\tau \right)$ (70), and $H^{M}\left(v\right)$, which are as follows: ${{\left\|H^{M}\left(v\right)\right\|}_{2}=\left\|H\left(\tau \right)\right\|}_{2}=19.257051\;Pa\cdot s^{1/2}$ and ${\left\|H\left(v\right)\right\|}_{2}=0.394490Pa\cdot s^{-1/2}$.

**Figure 12:**
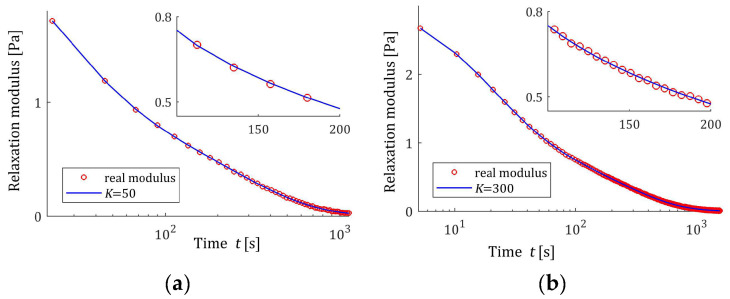
The measurements $\bar{G}\left(t_{k}\right)$ of double-mode Gauss-like time relaxation modulus $G\left(t\right)$ (72) corrupted by additive independent noises uniformly distributed over the interval $\left[-0.005,\;0.005\right]\;Pa$ (red points) and the corresponding relaxation modulus models ${\bar{G}}_{K}\left(t\right)$ (57) for K measurements of the relaxation modulus: (**a**) K=50; (**b**) K=300.

Based on the course of the modulus $G\left(t\right)$ (72), in the preliminary experiment, N=5000 sampling instants were generated with the constant period in the time interval $T=\left[0,1550\right]$ s, c.f., [27,28]. Following [27,28], additive measurement noises $z\left(t_{i}\right)$ were selected independently by random choice with uniform distribution on the interval $\left[-0.005,\;0.005\right]\;Pa$. The same as before, several K relaxation spectra models were determined using the proposed identification algorithm. The values of selected regularization parameters λ, the norms ${\left\|{\bar{H}}_{K}^{M}\left(v\right)\right\|}_{2}={\left\|{\bar{H}}_{K}\left(\tau \right)\right\|}_{2}$, ${\left\|{\bar{H}}_{K}\left(v\right)\right\|}_{2}$, and ${\left\|{\bar{g}}_{K}\left(\lambda \right)\right\|}_{2}$ and the indices $J\left({\bar{g}}_{K}\left(\lambda \right)\right)$, $J_{rel}\left({\bar{g}}_{K}\left(\lambda \right)\right)$, and $Q_{K}\left({\bar{g}}_{K}\left(\lambda \right)\right)$ (58) are presented in Table 4. The optimal models ${\bar{H}}_{K}^{M}\left(v\right)$ (41), ${\bar{H}}_{K}\left(v\right)$ (42), and ${\bar{H}}_{K}\left(\tau \right)$ (43) are depicted in Figure 9, Figure 10 and Figure 11 together with the real spectra plotted by red lines. The respective relaxation modulus models ${\bar{G}}_{K}\left(t\right)$ (57) are plotted in Figure 12 with the modulus $G\left(t\right)$ (72) measurements. 

However, for double-mode spectrum, the relative spectrum approximation index $J_{rel}\left({\bar{g}}_{K}\left(\lambda \right)\right)$ is as much as 25%, an inspection of Figure 9, Figure 10 and Figure 11 indicates a satisfactory approximation of the real spectra while maintaining the locations of both their maxima. Excellent models ${\bar{G}}_{K}\left(t\right)$ fit is confirmed by the values $Q_{K}\left({\bar{g}}_{K}\left(\lambda \right)\right)$ and Figure 12. 

### 3.10. Identification of KWW Relaxation Spectrum

The relaxation spectrum of the KWW model of the stretched exponential relaxation is described by the following [56]:

(73) $$G\left(t\right)=G_{0}e^{-{\left(\frac{t}{\tau_{r}}\right)}^{\beta }},$$

where the stretching exponent 0<β<1, $\tau_{r}$ is the relaxation time, and $G_{0}$ is the initial shear modulus, which has a unimodal [57] relaxation spectrum described by the infinite series [56,57]:

(74) $$H\left(\tau \right)=\frac{G_{0}}{\pi }\;\sum_{k=1}^{\infty }\frac{{\left(-1\right)}^{k+1}}{k!}\sin\left(\pi \beta k\right)\;\Gamma \left(\beta k+1\right)\;{\left(\frac{\tau }{\tau_{r}}\right)}^{\beta k},$$

where $\Gamma \left(n\right)$ is Euler’s gamma function ([62] Equation (8.310.1)). However, for the stretching exponent β=0.5, spectrum $H\left(\tau \right)$ has simple analytical form [57]:

(75) $$H\left(\tau \right)=\frac{G_{0}}{2\sqrt{\pi }}\sqrt{\frac{\tau }{\tau_{r}}}\;e^{-\frac{\tau }{4\tau_{r}\;}}.$$

**Figure 13:**
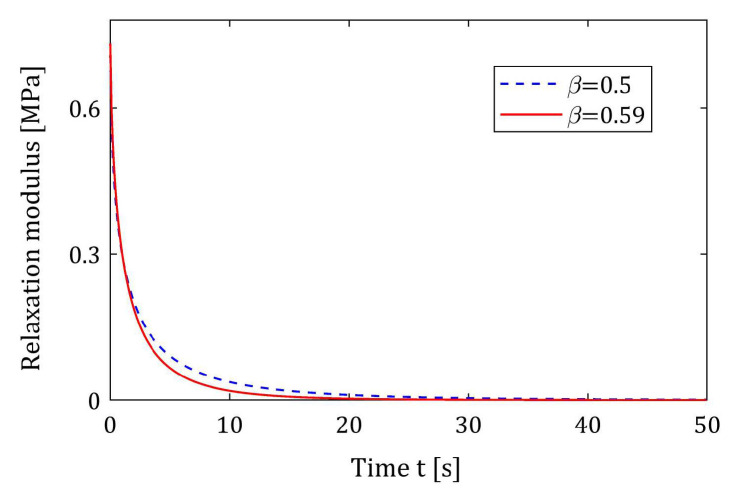
The KWW relaxation modulus $G\left(t\right)$ (73) for stretching exponents β=0.5 and β=0.59; the initial shear modulus $G_{0}=0.78\;MPa$; and the relaxation time $\tau_{r}=1.08\;s$.

The stretching exponent 0.5 is assumed, for which the relaxation spectrum is given by the analytical formula because the effectiveness of the identification method can only be verified when the assumed spectrum is exactly known. The exponent β=0.5 has been reported by Plazek and Ngai [63] for poly(methylphenylsiloxane) at the glass temperature $T_{g}=200\;K$; however, the related relaxation time $\tau_{r}$ is not reported in [63]. In 1993, Böhmer et al. [64], based on the literature and also private communications, have presented data concerning stretched exponential relaxation from about 70 amorphous polymeric glass formers (supercooled liquids and disordered crystals). The exponent β=0.5 has been experimentally obtained for sorbitol, dehydroabietic acid, BBKDE, 1,4-cis-polyisoprene, and silicate flint glass ([64] (Table I)). The coefficients β near 0.5 have been found for toluene (25%) (β=0.52), BCDE (β=0.51), polyisobutylene (β=0.55), and several other forms of glass. However, this paper also does not contain data on the related relaxation times. Recently, Chen et al. [65] applied the KWW model to describe the viscoelastic properties of the cross-linked polystyrene estimating the following parameters of the model (73) at temperature 110 °C: $G_{0}=0.78\;MPa$, β=0.59, and $\tau_{r}=1.08\;s$ ([65] (Table 4)). For the purpose of numerical tests of the algorithm, β=0.59 was replaced here by β=0.5. The discrepancy between the relaxation modulus $G\left(t\right)$ (73) for β=0.5 and β=0.59 is illustrated in Figure 13; the mean least-squares error for 500 equidistant sampling points between these modulus is equal to 1.2853 × 10^−4^ MPa^2^.

The relaxation frequency spectrum corresponding to (75) is as follows

(76) $$H\left(v\right)=\frac{G_{0}}{2\sqrt{\pi }}\sqrt{\frac{1}{\tau_{r}\cdot v}}\;e^{-\frac{1}{4\;\tau_{r}\cdot v}},$$

while the modified spectrum is described by the following:

(77) $$H^{M}\left(v\right)=\frac{G_{0}}{2\sqrt{\pi }}\sqrt{\frac{1}{\tau_{r}\cdot v^{3}}}\;e^{-\frac{1}{4\;\tau_{r}\cdot v}}.$$

**Figure 14:**
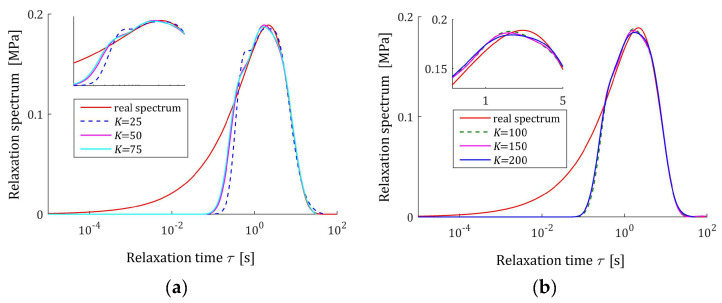
The KWW spectrum $H\left(\tau \right)$ (75) (solid red line) and the corresponding models ${\bar{H}}_{K}\left(\tau \right)$ (43) for K measurements of the relaxation modulus corrupted by additive independent noises uniformly distributed over the interval $\left[-0.5,\;0.5\right]\;kPa$: (**a**) K=25, 50, 75; (**b**) K=100, 150, 200.

**Figure 15:**
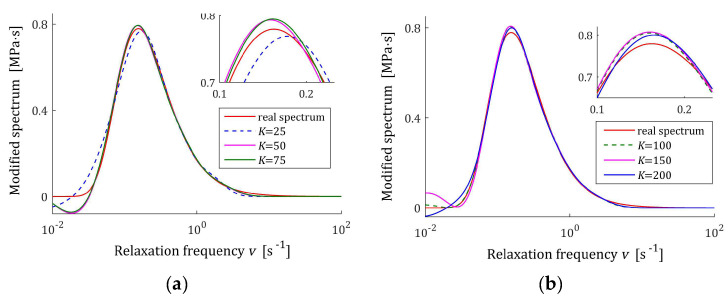
Modified KWW spectrum $H^{M}\left(v\right)$ (77) (solid red line) and the corresponding models ${\bar{H}}_{K}^{M}\left(v\right)$ (41) for K measurements of the relaxation modulus corrupted by additive independent noises uniformly distributed over the interval $\left[-0.5,\;0.5\right]\;kPa$: (**a**) K=25, 50, 75; (**b**) K=100, 150, 200.

**Figure 16:**
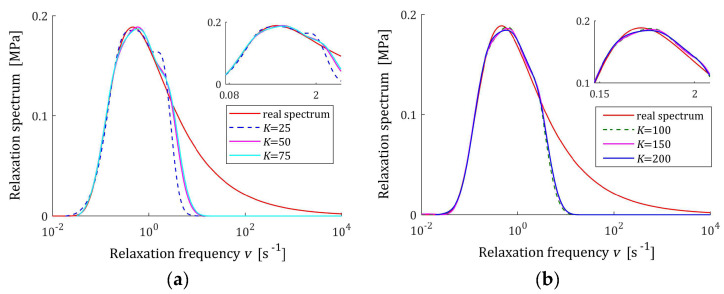
The KWW spectrum relaxation frequency spectrum $H\left(v\right)$ (76) (solid red line) and the corresponding models ${\bar{H}}_{K}\left(v\right)$ (42) for K measurements of the relaxation modulus corrupted by additive independent noises uniformly distributed over the interval $\left[-0.5,\;0.5\right]\;kPa$: (**a**) K=25, 50, 75; (**b**) K=100, 150, 200.

**Figure 17:**
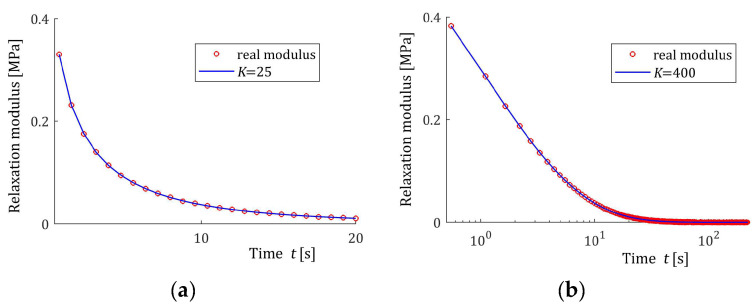
The measurements $\bar{G}\left(t_{k}\right)$ of the KWW relaxation modulus $G\left(t\right)$ (73) corrupted by additive independent noises uniformly distributed over the interval $\left[-0.5,\;0.5\right]\;kPa$ (red points) and the corresponding relaxation modulus models ${\bar{G}}_{K}\left(t\right)$ (57) for K measurements of the relaxation modulus: (**a**) K=25; (**b**) K=400.

In the preliminary experiment, N=500 sampling instants were generated with the constant period in the time interval $T=\left[0,50\right]$ s, selected based on the course of the modulus $G\left(t\right)$ (73) in Figure 13. Additive measurement noises $z\left(t_{i}\right)$ were selected independently by random choice with uniform distribution on the interval $\left[-0.5,\;0.5\right]\;kPa$. The values of the time scale factors α selected for a few values of K and the regularization parameters λ selected according to the rule (60) are given in Table 5. The best models ${\bar{H}}_{K}^{M}\left(v\right)$ (41), ${\bar{H}}_{K}\left(v\right)$ (42), and ${\bar{H}}_{K}\left(\tau \right)$ (43) are depicted in Figure 14, Figure 15 and Figure 16 together with the real spectra (red lines). The respective models ${\bar{G}}_{K}\left(t\right)$ (57) are plotted in Figure 17, with the modulus $G\left(t\right)$ (73) measurements. In Table 5, the norms ${\left\|{\bar{H}}_{K}^{M}\left(v\right)\right\|}_{2}={\left\|{\bar{H}}_{K}\left(\tau \right)\right\|}_{2}$, ${\left\|{\bar{H}}_{K}\left(v\right)\right\|}_{2}$, and the norms ${\left\|{\bar{g}}_{K}\left(\lambda \right)\right\|}_{2}$ of the optimal parameters ${\bar{g}}_{K}\left(\lambda \right)$ (39) are given. Since from (75) and (A3) we have the following:

$${\left\|H\left(\tau \right)\right\|}_{2}^{2}=\frac{G_{0}^{2}}{4\pi \;\tau_{r}}\int_{0}^{\infty }\tau e^{-\frac{\tau }{2\tau_{r}\;}}d\tau =\frac{G_{0}^{2}\tau_{r}}{\pi \;},$$

the norm ${\left\|H\left(\tau \right)\right\|}_{2}={\left\|H^{M}\left(v\right)\right\|}_{2}=G_{0}\sqrt{\tau_{r}/\pi \;}=0.457331\;MPa\cdot s^{1/2}$. In turn, the norm of the relaxation frequency spectrum (76) is infinite; however, in Table 5, the model’s norms ${\left\|{\bar{H}}_{K}\left(v\right)\right\|}_{2}$ are given. The integral indices $J\left({\bar{g}}_{K}\left(\lambda \right)\right)$ (63), the relative index $J_{rel}\left({\bar{g}}_{K}\left(\lambda \right)\right)$, (64) and the mean square relaxation modulus approximation index $Q_{K}\left({\bar{g}}_{K}\left(\lambda \right)\right)$ (58) are also presented in Table 5. 

The relative integral square index of the spectra approximation $J_{rel}\left({\bar{g}}_{K}\left(\lambda \right)\right)$ does not exceed 0.5% for K≥50 measurements, which means a better approximation of the assumed relaxation spectrum in the whole range of time/frequency relaxation variation, i.e., from zero to infinity, than in the case of Gaussian spectra. Also related to the relaxation modulus index, $Q_{K}\left({\bar{g}}_{K}\left(\lambda \right)\right)$, not exceeding 10^−7^, confirms the perfect approximation of the relaxation modulus measurements. In the case of this unimodal spectrum, increasing the number of measurements, i.e., the components of the series that create the models, does not significantly affect the quality of these models, which, in addition to the indices in Table 5, is also confirmed by a review of Figure 14, Figure 15 and Figure 16. For K≥100, the courses of the spectra models for increasing K remain practically almost identical, although a slight improvement in the fit to the real spectra can be seen in the values of the indices $J\left({\bar{g}}_{K}\left(\lambda \right)\right)$ and $J_{rel}\left({\bar{g}}_{K}\left(\lambda \right)\right)$. The relative index of the spectrum $H\left(\tau \right)$ approximation for K≥100 falls below 0.41%. 

### 3.11. Applicability of the Approach for Identification of Relaxation Spectra of Different Types

The rough condition of the approach’s successful applicability follows from the boundary properties of the optimal models ${\bar{H}}_{K}\left(\tau \right)$ (43) and ${\bar{H}}_{K}\left(v\right)$ (42), yielded by the properties of the basis functions $h_{k}\left(\tau \right)$ (15) and $h_{k}\left(v\right)v$, where $h_{k}\left(v\right)$ is given by (7). Since for $\tau \to 0^{+}$ and τ→∞, the basis functions $h_{k}\left(\tau \right)\to 0$, the best model ${\bar{H}}_{K}\left(\tau \right)$ (43) also tends to zero as the relaxation time τ tends to zero and to infinity, which limit the scope of applicability of this model to real relaxation time spectra that satisfy zero boundary conditions. For the relaxation frequencies v=0 and v→∞, the basis functions $h_{k}\left(v\right)v\to 0$, that is the basis functions $h_{k}\left(v\right)v$ of the relaxation frequency model ${\bar{H}}_{K}\left(v\right)$ (42) also have zero boundary conditions. Therefore, in terms of the relaxation frequency, the scope of applicability of the model and method to real relaxation frequency spectra is confined to the spectra of zero boundary conditions, too. 

The real relaxation time and frequency spectra and the known spectra models tend to zero as the relaxation time τ and the relaxation frequency v tend to infinity. Therefore, the properties of the spectra for $\tau \to 0^{+}$ and $v\to 0^{+}$ are essential here. 

The examples presented above showed that the approach proposed can be applied for Gauss-like relaxation spectra, both uni- and double mode, and for the KWW spectrum of the stretching exponent β=0.5. However, it is easy to check that for the relaxation spectrum $H\left(\tau \right)$ (74) both zero boundary conditions are satisfied. Therefore, the proposed identification method can be applied to determine the spectrum of materials whose relaxation processes have KWW stretched exponential nature. This is also important that the optimal model ${\bar{H}}_{K}\left(\tau \right)$ (43), given by a finite series, may prove to be more useful than the original KWW infinite series spectrum (74) for many applications. 

A multiplicative model that combines the power law with the stretched exponential relaxation described by Equation (8) in [66]:

(78) $$H\left(\tau \right)=n_{\alpha }G_{c}{\left(\frac{\tau }{\tau_{\alpha }}\right)}^{n_{\alpha }}\;e^{-{\left(\frac{\tau }{\tau_{\alpha }}\right)}^{\beta }},$$

where $\tau_{\alpha }$ is the longest relaxation time, $G_{c}$ is the plateau modulus, the stretching parameter 0<β≤1, and the exponent $0<n_{\alpha }<1$, was applied for modeling spectrum of bitumen in the vicinity of the glass transition [66]. The unimodal spectrum (78), named by the authors as the broadened power-law spectrum model [66], satisfies both zero boundary conditions—compare to ([66] (Figure 11a))—and therefore, is also within the scope of the proposed algorithm’s applicability.

However, the well-known Baumgaertel, Schausberger, and Winter (BSW) spectrum [15,67] used to describe the viscoelasticity of polybutadiene (PBD) [68], polydisperse polymer melts [8], polymethylmethacrylate (PMMA) [68], and many other materials, is described by the following model:

$$H\left(\tau \right)=\left\{\beta_{1}{\left(\frac{\tau }{\tau_{c}}\right)}^{\rho_{1}}+\beta_{2}{\left(\frac{\tau }{\tau_{c}}\right)}^{\rho_{2}}\right\}e^{-\frac{\tau }{\tau_{max}}},$$

with positive coefficients $\beta_{1}$, $\beta_{2}$ and relaxation times $\tau_{c}$, $\tau_{max}$, which tends to infinity for τ→0 whenever at least one of the parameters $\rho_{1}$ and $\rho_{2}$ is negative. Therefore, this is the case for real material models, compare [8,15,67,68], when the optimal model ${\bar{H}}_{K}\left(\tau \right)$ (43) cannot well-approximate this spectrum.

Likewise, the real relaxation spectra modeled by pure inverse power laws [69], for example, a combined four-interval power model with fractional exponents describing a solution-polymerized styrene butadiene rubber [70] or a power type spectrum with an exponent of −1/2 describing the cross-linking polymers at their gel point [71], cannot be successfully identified by the proposed approach. The relaxation time spectra of the fractional Maxwell model and the elementary fractional Scott–Blair model also lose the zero boundary condition at zero relaxation time, see [61] (Proposition 2, Equation (19)). 

### 3.12. Direct Identification of the Relaxation Spectra of Viscoelastic Solid Materials

For isotropic viscoelastic solids [31]

$$\underset{t\to \infty }{\lim}G\left(t\right)=G_{\infty }>0,$$

where $G_{\infty }$ is the material equilibrium modulus. Then, Equation (1) takes the form presented below [31]:

(79) $$G\left(t\right)=\int_{0}^{\infty }\frac{H\left(\tau \right)}{\tau }e^{-t/\tau }d\tau +G_{\infty }.$$

Analogously, Equation (5)—basic for the direct approach and related to the modified frequency spectrum $H^{M}\left(v\right)$—can be rewritten as follows:

(80) $$G\left(t\right)=\int_{0}^{\infty }H^{M}\left(v\right)e^{-tv}dv+G_{\infty }.$$

The relaxation spectra models $H_{K}^{M}\left(v\right)$ (9), $H_{K}\left(v\right)$ (10), and $H_{K}\left(\tau \right)$ (14) do not require modification, while the related relaxation modulus model $G_{K}\left(t\right)$ (11) should be replaced by the following:

$$G_{K}\left(t\right)=\int_{0}^{\infty }H_{K}^{M}\left(v\right)e^{-tv}dv+G_{\infty }=\sum_{k=1}^{K}g_{k}\phi_{k}\left(t\right)+G_{\infty },$$

which, however, does not affect the identification procedure itself. 

The square integral index $J\left(g_{K}\right)$, given by Equation (16) for the model $H_{K}^{M}\left(v\right)$ and by (20) for $H_{K}\left(\tau \right)$, is defined as above. However, by (80) and (7) we have:

(81) $$\int_{0}^{\infty }H^{M}\left(v\right)h_{k}\left(v\right)dv=\int_{0}^{\infty }H^{M}e^{-\alpha kv}dv=G\left(\alpha k\right)-G_{\infty }=∆G\left(\alpha k\right).$$

Therefore, by (17) and (81), the index $J\left(g_{K}\right)$ is given by the following expression (compare to (19)):

$$J\left(g_{K}\right)=\int_{0}^{\infty }{H^{M}\left(v\right)}^{2}dv-2\sum_{k=1}^{K}g_{k}∆G\left(\alpha k\right)+\sum_{k=1}^{K}{\sum_{m=1}^{K}g_{k}g}_{m}\varphi_{km}.$$

As above, from (79) and (20), the analogous formula results for the relaxation time spectrum model. As a consequence, the integral-empirical index ${\bar{J}}_{K}\left(g_{K}\right)$ (24) is now as follows

$${\bar{J}}_{K}\left(g_{K}\right)=\int_{0}^{\infty }{H^{M}\left(v\right)}^{2}dv-2\sum_{k=1}^{K}g_{k}∆\bar{G}\left(\alpha k\right)+\sum_{k=1}^{K}{\sum_{m=1}^{K}g_{k}g}_{m}\varphi_{km},$$

where, compare $∆G\left(\alpha k\right)$ (81), the relaxation modulus increment is defined as follows:

(82) $$∆\bar{G}\left(\alpha k\right)=\bar{G}\left(\alpha k\right)-G_{\infty }.$$

Since real materials may relax over a very long time, two cases can occur.

*Case 1*. If the duration of the relaxation test can be extended so as to experimentally record a time-constant relaxation modulus (in practice, constant stress), then $G_{\infty }$ is experimentally evaluated and the proposed identification algorithm can be simply applied by replacing the measurements $\bar{G}\left(\alpha k\right)$ with their increments $∆\bar{G}\left(\alpha k\right)$ (82) in relation to known $G_{\infty }$.
*Case 2*. For identification purposes, only time-varying relaxation modulus measurements are available, i.e., the steady-state stress was not recorded during the experiment. In such a situation, non-negative $G_{\infty }$ is an additional model parameter that should be extrapolated beyond the experiment time horizon limited by the upper bound $t_{K}=\alpha K$. The linear-quadratic problem (32) of optimal identification needs to be reformulated, re-regularized and solved, which creates a new research problem.

However, for many materials, the equilibrium modulus is accessible by experiment; then, the algorithm of direct relaxation spectra identification can be applied with the simple modification as described above.

## 4. Conclusions

Summarizing, this paper addresses the relaxation spectrum identification problem in a new original way. The novelty of the paper is that it directly takes into account the unknown spectrum in the model quality index being minimized. The main result is based only on the definition of the relaxation spectrum, which relates the spectrum to the measurable relaxation modulus, and on the fact that the set of exponential functions, i.e., a kernel of the Lagrange transform constitute a basis of the space of square-integrable functions. The analytical and numerical studies demonstrated that by applying the proposed relaxation spectra models and identification algorithm, it is possible to determine the spectra models for a wide range of relaxation times and frequencies of real materials. 

The concept of direct relaxation spectrum identification can be applied both for viscoelastic fluids and viscoelastic solids; however, for solid materials, a respective modification of the algorithm may be required whenever the equilibrium relaxation modulus is not available by measurement, the development of which will be the subject of further research.

It is generally accepted that the choice of respective regularization parameters is important to identify the best model. The well-studied techniques for computing a good regularization parameter such as the discrepancy principle, generalized cross-validation, and the L-curve technique have been developed for classical least-squares task and hence they cannot be directly applied here. Therefore, the regularized minimization problem (32) should be reformulated to the classic form of the linear least-squares problem. Then, the applicability of the known techniques can be verified. An alternative approach is to develop a new method of selecting the regularization parameter, specifically addressing the problem of direct spectrum identification. Although the numerical studies have shown that the simple rule based on the condition number of the basic matrix for the linear-quadratic identification problem is sufficient in many cases, the example of a two-mode Gaussian-like spectrum motivates the search for a better rule for the regularization parameter selection, dedicated for this specific identification task. This will be the subject of further research.

The impact of the molecular weight distributions (MWD) on the viscoelastic properties is intensively studied in polymer rheology. Generic analytical formulas describing the relationship between MWD and the relaxation time spectrum are known. Future research directions may include the determination of the MWD, which can be obtained from the relaxation time spectrum model and recovered from experimental results by the proposed method.

## Figures and Tables

**Table 1:** Spectral condition numbers $\kappa \left(\Phi_{K}\right)$ and $\kappa \left(\Phi_{K}+\alpha \lambda I_{K,K}\right)$ defined according to (30).

αλ	K = 2	K = 3	K = 4	K = 5	K = 8	K = 10	K = 15	K = 20	K = 100	K = 1000	K = 10,000
0	38.474	1.35 × 10^3^	4.59 × 10^4^	1.54 × 10^6^	5.64 × 10^10^	6.23 × 10^13^	2.61 × 10^17^	6.45 × 10^18^	1.20 × 10^19^	9.59 × 10^20^	1.0 × 10^22^
10	1.071	1.087	1.098	1.106	1.122	1.129	1.14129	1.1496	1.18809	1.225	1.248
1	1.699	1.874	1.977	2.056	2.215	2.288	2.412	2.495	2.880	3.247	3.479
0.1	6.983	9.688	10.773	11.559	13.154	13.877	15.123	15.953	19.800	23.469	25.798
0.01	25.552	83.135	98.55	106.59	122.542	129.766	142.232	150.535	189.001	225.687	248.977
1 × 10^−3^	36.600	532.056	958.14	1.06 × 10^3^	1.22 × 10^3^	1.29 × 10^3^	1.41 × 10^3^	1.49 × 10^3^	1.88 × 10^3^	2.25 × 10^3^	2.48 × 10^3^
1 × 10^−4^	38.278	1.17 × 10^3^	8.06 × 10^3^	1.05 × 10^4^	1.22 × 10^4^	1.29 × 10^4^	1.41 × 10^4^	1.49 × 10^4^	1.88 × 10^4^	2.25 × 10^4^	2.48 × 10^4^
1 × 10^−5^	38.454	1.33× 10^3^	3.12 × 10^4^	9.88 × 10^4^	1.22 × 10^5^	1.29 × 10^5^	1.41 × 10^5^	1.49 × 10^5^	1.88 × 10^5^	2.25 × 10^5^	2.48 × 10^5^

**Table 2:** The square roots of the largest $\sigma_{1}$, $\varsigma_{1}$ and minimal $\sigma_{K}$, $\varsigma_{K}$ singular value of the matrices $\Phi_{K}$ (25) and $\Theta_{K}$ (46) for K model summands.

K	10	50	100	500	1000	5000	10,000
$\sqrt{\sigma_{1}}$	1.1348	1.3153	1.3711	1.4677	1.4989	1.5556	1.5747
$\sqrt{\sigma_{K}}$	1.437 × 10^−7^	1.919 × 10^−10^	3.955 × 10^−10^	1.923 × 10^−10^	4.838 × 10^−11^	2.323 × 10^−11^	1.573 × 10^−11^
$\sqrt{\varsigma_{1}}$	0.3737	0.3737	0.3737	0.3737	0.3737	0.3737	0.3737
$\sqrt{\varsigma_{K}}$	6.829 × 10^−8^	1.151 × 10^−11^	1.847 × 10^−12^	1.017 × 10^−13^	2.455 × 10^−14^	2.769 × 10^−15^	6.473 × 10^−15^

**Table 3:** For the uni-mode Gauss-like spectrum, $H\left(\tau \right)$ (65), and the models ${\bar{H}}_{K}^{M}\left(v\right)$ (41),$\;{\bar{H}}_{K}\left(v\right)$ (42), and ${\bar{H}}_{K}\left(\tau \right)$ (43): time-scale factors α; numbers of model summands K; regularization parameters λ; the model’s smoothness indices ${\left\|{\bar{H}}_{K}^{M}\left(v\right)\right\|}_{2}={\left\|{\bar{H}}_{K}\left(\tau \right)\right\|}_{2}$ (61) and ${\left\|{\bar{H}}_{K}\left(v\right)\right\|}_{2}$ (62); the mean square relaxation modulus approximation index $Q_{K}\left({\bar{g}}_{K}\left(\lambda \right)\right)$ (58); norms ${\left\|{\bar{g}}_{K}\left(\lambda \right)\right\|}_{2}$ (49) of the model parameter vectors; the integral square approximation indices $J\left({\bar{g}}_{K}\left(\lambda \right)\right)$ (63); and relative index $J_{rel}\left({\bar{g}}_{K}\left(\lambda \right)\right)$ (64).

K	α [s]	λ $\left[s^{-1}\right]$	${\left\|{\bar{H}}_{K}\left(\tau \right)\right\|}_{2}$ $\left[kPa\cdot s^{1/2}\right]$	${\left\|{\bar{H}}_{K}\left(v\right)\right\|}_{2}$ $\left[kPa\cdot s^{-1/2}\right]$	$Q_{K}\left({\bar{g}}_{K}\left(\lambda \right)\right)$ $\left[{kPa}^{2}\right]$	${\left\|{\bar{g}}_{K}\left(\lambda \right)\right\|}_{2}$ $\left[kPa\cdot s\right]$	$J\left({\bar{g}}_{K}\left(\lambda \right)\right)$ $\left[{kPa}^{2}\cdot s\right]$	$J_{rel}\left({\bar{g}}_{K}\left(\lambda \right)\right)$
20	4.90	3.1 × 10^−6^	8.194688	1.000985	3.681218 × 10^−5^	8.752841 × 10^3^	3.548595	0.0500
50	3.75	9.0 × 10^−6^	8.167268	0.847115	3.486716 × 10^−5^	4.639282 × 10^3^	2.461727	0.0347
100	3.98	7.8 × 10^−6^	8.193556	0.861458	3.272773 × 10^−5^	7.334379 × 10^3^	2.439776	0.0344
150	3.75	5.5 × 10^−6^	8.248417	0.865259	3.310334 × 10^−5^	1.281206 × 10^4^	2.625005	0.0370
200	4.08	9.5 × 10^−6^	8.173553	0.869989	3.299329 × 10^−5^	8.550751 × 10^3^	2.875342	0.0405

**Table 4:** For the double-mode Gauss-like spectrum $H\left(\tau \right)$ (70) and the models ${\bar{H}}_{K}^{M}\left(v\right)$ (41),$\;{\bar{H}}_{K}\left(v\right)$ (42) and ${\bar{H}}_{K}\left(\tau \right)$ (43): time-scale factors α; numbers of model summands K; regularization parameters λ; the model’s smoothness indices ${\left\|{\bar{H}}_{K}^{M}\left(v\right)\right\|}_{2}={\left\|{\bar{H}}_{K}\left(\tau \right)\right\|}_{2}$ and ${\left\|{\bar{H}}_{K}\left(v\right)\right\|}_{2}$; the mean square relaxation modulus approximation index $Q_{K}\left({\bar{g}}_{K}\left(\lambda \right)\right)$ (58); norms ${\left\|{\bar{g}}_{K}\left(\lambda \right)\right\|}_{2}$ (49) of the model parameter vectors; the integral square approximation indices $J\left({\bar{g}}_{K}\left(\lambda \right)\right)$ (63); and relative index $J_{rel}\left({\bar{g}}_{K}\left(\lambda \right)\right)$ (64).

K	α [s]	λ $\left[s^{-1}\right]$	${\left\|{\bar{H}}_{K}\left(\tau \right)\right\|}_{2}$ $\left[Pa\cdot s^{1/2}\right]$	${\left\|{\bar{H}}_{K}\left(v\right)\right\|}_{2}$ $\left[Pa\cdot s^{-1/2}\right]$	$Q_{K}\left({\bar{g}}_{K}\left(\lambda \right)\right)$ $\left[{Pa}^{2}\right]$	${\left\|{\bar{g}}_{K}\left(\lambda \right)\right\|}_{2}$ $\left[Pa\cdot s\right]$	$J\left({\bar{g}}_{K}\left(\lambda \right)\right)$ $\left[{Pa}^{2}\cdot s\right]$	$J_{rel}\left({\bar{g}}_{K}\left(\lambda \right)\right)$
50	22.5	8 × 10^−7^	17.008811	0.377417	8.91256 × 10^−6^	2.63874 × 10^4^	83.212916	0.224394
100	16.3	1.2 × 10^−6^	16.749729	0.415414	8.20297 × 10^−6^	2.38674 × 10^4^	89.54064	0.241457
150	9.35	2.1 × 10^−6^	16.145506	0.440330	8.33036 × 10^−6^	1.68329 × 10^4^	90.59845	0.244309
200	6.5	3.1 × 10^−6^	16.447062	0.404095	8.38748 × 10^−6^	1.321209 × 10^4^	85.71129	0.2311311
300	5.2	4 × 10^−6^	16.650568	0.401779	8.04576 × 10^−6^	1.228249 × 10^4^	87.92225	0.237093

**Table 5:** For the KWW spectrum $H\left(\tau \right)$ (75) and the models ${\bar{H}}_{K}^{M}\left(v\right)$ (41), ${\bar{H}}_{K}\left(v\right)$ (42), and ${\bar{H}}_{K}\left(\tau \right)$ (43): time-scale factors α; numbers of model summands K; regularization parameters λ; the model’s smoothness indices ${\left\|{\bar{H}}_{K}^{M}\left(v\right)\right\|}_{2}={\left\|{\bar{H}}_{K}\left(\tau \right)\right\|}_{2}$ and ${\left\|{\bar{H}}_{K}\left(v\right)\right\|}_{2}$; the mean square relaxation modulus approximation index $Q_{K}\left({\bar{g}}_{K}\left(\lambda \right)\right)$ (58); norms ${\left\|{\bar{g}}_{K}\left(\lambda \right)\right\|}_{2}$ (49) of the model parameter vectors; the integral square approximation indices $J\left({\bar{g}}_{K}\left(\lambda \right)\right)$ (63); and relative index $J_{rel}\left({\bar{g}}_{K}\left(\lambda \right)\right)$ (64).

K	α[s]	$\lambda [s^{-1}]$	${\left\|{\bar{H}}_{K}\left(\tau \right)\right\|}_{2}[MPa\cdot s^{1/2}]$	${\left\|{\bar{H}}_{K}\left(v\right)\right\|}_{2}\left[MPa\cdot s^{-1/2}\right]$	$Q_{K}\left({\bar{g}}_{K}\left(\lambda \right)\right)\left[M{Pa}^{2}\right]$	${\left\|{\bar{g}}_{K}\left(\lambda \right)\right\|}_{2}\left[MPa\cdot s\right]$	$J\left({\bar{g}}_{K}\left(\lambda \right)\right)\left[{MPa}^{2}\cdot s\right]$	$J_{rel}\left({\bar{g}}_{K}\left(\lambda \right)\right)$
25	0.8	2 × 10^−5^	0.454723	0.267896	9.46218 × 10^−8^	76.901664	1.83447 × 10^−3^	8.77098 × 10^−3^
50	0.65	7 × 10^−5^	0.456956	0.284479	8.70335 × 10^−8^	29.800961	9.82909 × 10^−4^	4.69948 × 10^−3^
75	0.6	7.5 × 10^−5^	0.4571396	0.289756	8.52022 × 10^−8^	33.705039	8.50259 × 10^−4^	4.06526 × 10^−3^
100	0.65	8.5 × 10^−5^	0.456765	0.284243	8.18454 × 10^−8^	33.6572199	8.26024 × 10^−4^	3.94938 × 10^−3^
150	0.6	1 × 10^−4^	0.457356	0.289670	8.28962 × 10^−8^	35.262493	8.41199 × 10^−4^	4.02194 × 10^−3^
200	0.6	1.5 × 10^−4^	0.456657	0.289207	8.25479 × 10^−8^	27.087999	7.34565 × 10^−4^	3.51210 × 10^−3^
300	0.55	1.6 × 10^−4^	0.456875	0.294931	7.97708 × 10^−8^	30.574734	7.12159 × 10^−4^	3.40497 × 10^−3^
400	0.55	1.6 × 10^−4^	0.456791	0.294901	8.10268 × 10^−8^	35.581507	7.12571 × 10^−4^	3.40694 × 10^−3^

## Data Availability

The original contributions presented in the study are included in the article; further inquiries can be directed to the corresponding author.

## Appendix A

### Appendix A.1. Proof of Lemma 1

According to (6), (17) and (25), the quadratic form $g_{K}^{T}\Phi_{K}g_{K}$ is expressed as follows:

$$g_{K}^{T}\Phi_{K}g_{K}=\alpha \sum_{k=1}^{K}\sum_{m=1}^{K}g_{k}g_{m}\frac{1}{\left(k+m\right)\alpha }=\alpha \int_{0}^{\infty }{\left[\sum_{k=1}^{K}g_{k}e^{-\alpha kv}\right]}^{2}dv.$$

Thus, $g_{K}^{T}\Phi_{K}g_{K}\geq 0$ for an arbitrary vector $g_{K}$, and $g_{K}^{T}\Phi_{K}g_{K}=0$, if and only if $\sum_{k=1}^{K}g_{k}e^{-\alpha kv}=0$ for almost all v>0. Since the basis functions $h_{k}\left(v,\alpha \right)=e^{-\alpha kv}$ are independent, the last equality holds, if and only if $g_{k}=0$ for all k=1,…,K, i.e., only if the vector $g_{K}=0$, which yields the positive definiteness of $\Phi_{K}$. This finishes the proof. □

### Appendix A.2. Proof of Proposition 1

For the model ${\bar{H}}_{K}^{M}\left(v\right)$ (41) of the modified spectrum $H^{M}\left(v\right)$ (4), by (18), we have the following:

(A1) $${\left\|{\bar{H}}_{K}^{M}\left(v\right)\right\|}_{2}^{2}=\int_{0}^{\infty }{\left[{\bar{H}}_{K}^{M}\left(v\right)\right]}^{2}dv=\sum_{k=1}^{K}{\sum_{m=1}^{K}{\bar{g}}_{k}\left(\lambda \right)g}_{m}\varphi_{km}=\frac{1}{\alpha }{\bar{g}}_{K}^{T}\left(\lambda \right)\Phi_{K}{\bar{g}}_{K}\left(\lambda \right),$$

with the vector of model parameters ${\bar{g}}_{K}\left(\lambda \right)$ (34). Similarly, (43) and (18), yield

(A2) $${\left\|{\bar{H}}_{K}\left(\tau \right)\right\|}_{2}^{2}=\int_{0}^{\infty }{\left[{\bar{H}}_{K}\left(\tau \right)\right]}^{2}d\tau =\sum_{k=1}^{K}\sum_{m=1}^{K}{\bar{g}}_{k}\left(\lambda \right){\bar{g}}_{m}\left(\lambda \right)\varphi_{km}=\;\frac{1}{\alpha }{\bar{g}}_{K}^{T}\left(\lambda \right)\Phi_{K}{\bar{g}}_{K}\left(\lambda \right).$$

By the following the integral formula ([62] Equation (3.351.3))

(A3) $$\int_{0}^{\infty }\tau^{n}e^{-\beta \tau }d\tau =\frac{n!}{\beta^{n+1}},$$

for the model ${\bar{H}}_{K}\left(v\right)$ (42) of the real spectrum $H\left(v\right)$ we have the following:

(A4) $${\left\|{\bar{H}}_{K}\left(v\right)\right\|}_{2}^{2}=\int_{0}^{\infty }{\left[{\bar{H}}_{K}\left(v\right)\right]}^{2}dv=\frac{2}{\alpha^{3}}\sum_{k=1}^{K}\sum_{m=1}^{K}{\bar{g}}_{k}\left(\lambda \right){\bar{g}}_{m}\left(\lambda \right)\frac{1}{{\left(k+m\right)}^{3}}=\;\frac{2}{\alpha^{3}}{\bar{g}}_{K}^{T}\left(\lambda \right)\Theta_{K}{\bar{g}}_{K}\left(\lambda \right),$$

where the K×K positive definite (the proof is analogous to that of Lemma 1) matrix $\Theta_{K}$ of the elements $\theta_{km}=\frac{1}{{\left(k+m\right)}^{3}}$ is defined by Equation (46). The equalities in (44) and (45) are proved.

According the known Rayeigh–Ritz inequalities [72], (Lemma I):

(A5) $$\lambda_{min}\left(X\right)x^{T}x\leq x^{T}Xx\leq \lambda_{max}\left(X\right)x^{T}x,$$

which holds for any $x\in R^{m}$ and any symmetric matrix $X=X^{T}\in R^{m,m}$, where $\lambda_{min}\left(X\right)$ and $\lambda_{max}\left(X\right)$ are minimal and maximal eigenvalues of the matrix X. Since for positive definite $\Phi_{K}$ and $\Theta_{K}$ their eigenvalues are identical to the singular values [39] (p. 77), in view of (A5) Equations (A1), (A2), and (A4), imply the lower and upper bounds in (44) and (45). Proposition is proved. □

### Appendix A.3. Proof of Proposition 3

The error between the spectra ${\bar{H}}_{K}^{M}\left(v\right)$ (41) and ${\tilde{H}}_{K}^{M}\left(v\right)$ (50) is given by the formula below:

$${\bar{H}}_{K}^{M}\left(v\right)-{\tilde{H}}_{K}^{M}\left(v\right)=\sum_{k=1}^{K}\left[{\bar{g}}_{k}\left(\lambda \right)-{\tilde{g}}_{k}\left(\lambda \right)\right]h_{k}\left(v\right),$$

therefore, the integral square error between these spectra is as follows:

$${\left\|{\bar{H}}_{K}^{M}\left(v\right)-{\tilde{H}}_{K}^{M}\left(v\right)\right\|}_{2}^{2}=\int_{0}^{\infty }{\left[\sum_{k=1}^{K}\left[{\bar{g}}_{k}\left(\lambda \right)-{\tilde{g}}_{k}\left(\lambda \right)\right]h_{k}\left(v\right)\right]}^{2}dv,$$

and, in view of (18), is described by the next formula:

$${\left\|{\bar{H}}_{K}^{M}\left(v\right)-{\tilde{H}}_{K}^{M}\left(v\right)\right\|}_{2}^{2}=\sum_{k=1}^{K}\sum_{m=1}^{K}\left[{\bar{g}}_{k}\left(\lambda \right)-{\tilde{g}}_{k}\left(\lambda \right)\right]\left[{\bar{g}}_{m}\left(\lambda \right)-{\tilde{g}}_{m}\left(\lambda \right)\right]\varphi_{km},$$

whence, having in mind the notation (25), the quadratic form is obtained

(A6) $${\left\|{\bar{H}}_{K}^{M}\left(v\right)-{\tilde{H}}_{K}^{M}\left(v\right)\right\|}_{2}^{2}=\frac{1}{\alpha }{\left[{\bar{g}}_{K}\left(\lambda \right)-{\tilde{g}}_{K}\left(\lambda \right)\right]}^{T}\Phi_{K}\left[{\bar{g}}_{K}\left(\lambda \right)-{\tilde{g}}_{K}\left(\lambda \right)\right].$$

By (51) and (39)

(A7) $${{\bar{g}}_{K}\left(\lambda \right)-\tilde{g}}_{K}\left(\lambda \right)=\alpha U_{K}\Omega_{K}U_{K}^{T}z_{N}$$

with the vector of the measurement noises $z_{N}={\bar{G}}_{K}-G_{K}$, which, substituted into (A6) and combined with the SVD (35), yields

$${\left\|{\bar{H}}_{K}^{M}\left(v\right)-{\tilde{H}}_{K}^{M}\left(v\right)\right\|}_{2}^{2}=\alpha z_{N}^{T}U_{K}\Omega_{K}\Sigma_{K}\Omega_{K}U_{K}^{T}z_{N}.$$

Diagonal structure of the matrices $\Sigma_{K}$ (36) and $\Omega_{K}$ (38) implies the structure of the next matrix

$$\Omega_{K}\Sigma_{K}\Omega_{K}=diag\left(\frac{\sigma_{1}}{{\left(\sigma_{1}+\alpha \lambda \right)}^{2}},\ldots ,\frac{\sigma_{K}}{{\left(\sigma_{K}+\alpha \lambda \right)}^{2}}\right),$$

whence, by the right inequality in (A5) and since for orthogonal $U_{K}$ we have $z_{N}^{T}U_{K}U_{K}^{T}z_{N}=z_{N}^{T}z_{N}$, the next upper bound is obtained

$${\left\|{\bar{H}}_{K}^{M}\left(v\right)-{\tilde{H}}_{K}^{M}\left(v\right)\right\|}_{2}^{2}\leq \alpha \underset{1\leq k\leq K}{\max}\frac{\sigma_{k}}{{\left(\sigma_{k}+\alpha \lambda \right)}^{2}}z_{N}^{T}z_{N},$$

whence the inequality (54) with parameter γ (55) for the models ${\bar{H}}_{K}^{M}\left(v\right)$ and ${\tilde{H}}_{K}^{M}\left(v\right)$ directly follows.

Similarly, for the spectra ${\bar{H}}_{K}\left(\tau \right)$ (43) and ${\tilde{H}}_{K}\left(\tau \right)$ (53) we have the following:

$${{\bar{H}}_{K}\left(\tau \right)-\tilde{H}}_{K}\left(\tau \right)=\sum_{k=1}^{K}\left[{\bar{g}}_{k}\left(\lambda \right)-{\tilde{g}}_{k}\left(\lambda \right)\right]h_{k}\left(\tau \right),$$

whence, having in mind (22), we obtain

$${\left\|{{\bar{H}}_{K}\left(\tau \right)-\tilde{H}}_{K}\left(\tau \right)\right\|}_{2}^{2}=\frac{1}{\alpha }{\left[{\bar{g}}_{K}\left(\lambda \right)-{\tilde{g}}_{K}\left(\lambda \right)\right]}^{T}\Phi_{K}\left[{\bar{g}}_{K}\left(\lambda \right)-{\tilde{g}}_{K}\left(\lambda \right)\right],$$

that is, this norm is identical to (A6); the second inequality in (54) follows.

Finally, for the spectra ${\bar{H}}_{K}\left(v\right)$ (42) and ${\tilde{H}}_{K}\left(v\right)$ (52) we have the following:

$${\bar{H}}_{K}\left(v\right)-{\tilde{H}}_{K}\left(v\right)=\sum_{k=1}^{K}\left[{\bar{g}}_{k}\left(\lambda \right)-{\tilde{g}}_{k}\left(\lambda \right)\right]h_{k}\left(v\right)v,$$

whence, having in mind the matrix $\Theta_{K}$ introduced in (A4), we obtain

$${\left\|{\bar{H}}_{K}\left(v\right)-{\tilde{H}}_{K}\left(v\right)\right\|}_{2}^{2}=\frac{2}{\alpha^{3}}{\left[{\bar{g}}_{K}\left(\lambda \right)-{\tilde{g}}_{K}\left(\lambda \right)\right]}^{T}\Theta_{K}\left[{\bar{g}}_{K}\left(\lambda \right)-{\tilde{g}}_{K}\left(\lambda \right)\right],$$

and next, by (A7),

$${\left\|{\bar{H}}_{K}\left(v\right)-{\tilde{H}}_{K}\left(v\right)\right\|}_{2}^{2}=\frac{2}{\alpha }z_{N}^{T}U_{K}\Omega_{K}U_{K}^{T}\Theta_{K}U_{K}\Omega_{K}U_{K}^{T}z_{N}.$$

By applying the right inequality in (A5) and including the orthogonality of $U_{K}$ we have:

$${\left\|{\bar{H}}_{K}\left(v\right)-{\tilde{H}}_{K}\left(v\right)\right\|}_{2}^{2}\leq \frac{2}{\alpha }\varsigma_{1}z_{N}^{T}U_{K}\Omega_{K}\Omega_{K}U_{K}^{T}z_{N}.$$

whence in view of the structure of the matrix $\Omega_{K}$ (38) we immediately obtain

$${\left\|{\bar{H}}_{K}\left(v\right)-{\tilde{H}}_{K}\left(v\right)\right\|}_{2}^{2}\leq \frac{2\;\varsigma_{1}}{\alpha {\left(\sigma_{K}+\alpha \lambda \right)}^{2}}z_{N}^{T}z_{N},$$

which implies (56) and completes the proof. □

### Appendix A.4. Proof of Proposition 4

Since for any $t_{k}=\alpha k$ and any m, by (12) and (18), we have the following:

$$\phi_{m}\left(t_{k}\right)=\frac{1}{\alpha k+\alpha m}=\varphi_{km}=\varphi_{mk},$$

the value of the relaxation modulus model ${\bar{G}}_{K}\left(t\right)$ (57) for $t=t_{k}=\alpha k$ can be described by the equation below:

$${\bar{G}}_{K}\left(t_{k}\right)=\sum_{m=1}^{K}{\bar{g}}_{m}\left(\lambda \right)\phi_{m}\left(t_{k}\right)=\sum_{m=1}^{K}{\bar{g}}_{m}\left(\lambda \right)\varphi_{km}=\sum_{m=1}^{K}{\bar{g}}_{m}\left(\lambda \right)\varphi_{mk}.$$

Therefore, index $Q_{K}\left({\bar{g}}_{K}\left(\lambda \right)\right)$ (58) can be expressed as follows:

$$Q_{K}\left({\bar{g}}_{K}\left(\lambda \right)\right)=\frac{1}{K}\sum_{k=1}^{K}{\left[\bar{G}\left(t_{k}\right)\right]}^{2}+\frac{1}{K}\sum_{k=1}^{K}\sum_{m=1}^{K}{\bar{g}}_{m}\left(\lambda \right)\varphi_{mk}\varphi_{km}{\bar{g}}_{k}\left(\lambda \right)-\;\frac{2}{K}\sum_{k=1}^{K}\sum_{m=1}^{K}{\bar{g}}_{m}\left(\lambda \right)\varphi_{mk}\bar{G}\left(t_{k}\right),$$

whence, due to (22) and (25), i.e., having in mind that elements of the matrix $\Phi_{K}$ are equal to $\alpha \varphi_{km}$, the equivalent matrix-vector form follows

$$Q_{K}\left({\bar{g}}_{K}\left(\lambda \right)\right)=\frac{1}{K}{\bar{G}}_{K}^{T}{\bar{G}}_{K}+\frac{1}{K}\frac{1}{\alpha^{2}}{\bar{g}}_{K}^{T}\left(\lambda \right)\Phi_{K}\Phi_{K}{\bar{g}}_{K}\left(\lambda \right)-\frac{2}{K}\frac{1}{\alpha }{\bar{G}}_{K}^{T}\Phi_{K}{\bar{g}}_{K}\left(\lambda \right),$$

which in compact form is given by the following:

$$Q_{K}\left({\bar{g}}_{K}\left(\lambda \right)\right)=\frac{1}{K}{\left[{\bar{G}}_{K}-\frac{1}{\alpha }\Phi_{K}{\bar{g}}_{K}\left(\lambda \right)\right]}^{T}\left[{\bar{G}}_{K}-\frac{1}{\alpha }\Phi_{K}{\bar{g}}_{K}\left(\lambda \right)\right].$$

The first equality in (59) is derived.

By the SVD (35), including Formula (39), the above can be rewritten as outlined below:

$$Q_{K}\left({\bar{g}}_{K}\left(\lambda \right)\right)=\frac{1}{K}{\left[{\bar{G}}_{K}-\frac{1}{\alpha }U_{K}\Sigma_{K}U_{K}^{T}U_{K}\Omega_{K}Y_{K}\right]}^{T}\left[{\bar{G}}_{K}-\frac{1}{\alpha }U_{K}\Sigma_{K}U_{K}^{T}U_{K}\Omega_{K}Y_{K}\right];$$

whence, due to orthogonality of $U_{K}$, we obtain

$$Q_{K}\left({\bar{g}}_{K}\left(\lambda \right)\right)=\frac{1}{K}\left[{\bar{G}}_{K}^{T}{\bar{G}}_{K}-{2Y}_{K}^{T}\Sigma_{K}\Omega_{K}Y_{K}+Y_{K}^{T}\Omega_{K}\Sigma_{K}\Sigma_{K}\Omega_{K}Y_{K}\right].$$

The diagonal structure of the matrices $\Sigma_{K}$ (36) and $\Omega_{K}$ (38) yields

$$\Sigma_{K}\Omega_{K}=diag\left(\frac{\sigma_{1}}{\sigma_{1}+\alpha \lambda },\ldots ,\frac{\sigma_{K}}{\sigma_{K}+\alpha \lambda }\right),$$

whence, remembering that $Y_{K}=U_{K}^{T}{\bar{G}}_{K}$ (40), we have the following:

$$Q_{K}\left({\bar{g}}_{K}\left(\lambda \right)\right)=\sum_{k=1}^{K}\left[1-\frac{{2\sigma }_{k}}{\sigma_{k}+\alpha \lambda }+\frac{{\left(\sigma_{k}\right)}^{2}}{{\left(\sigma_{k}+\alpha \lambda \right)}^{2}}\right]y_{k}^{2},$$

and, after algebraic manipulations, equivalently,

$$Q_{K}\left({\bar{g}}_{K}\left(\lambda \right)\right)=\sum_{k=1}^{K}\frac{{\left(\alpha \lambda \right)}^{2}y_{k}^{2}}{{\left(\sigma_{k}+\alpha \lambda \right)}^{2}}.$$

whence second Equation in (59) directly follows. □

### Appendix A.5. Norms of the Spectra $H\left(\tau \right)$ (65), $H\left(v\right)$ (68), and $H^{M}\left(v\right)$ (69)

By (69),

$${\left\|H^{M}\left(v\right)\right\|}_{2}^{2}=\int_{0}^{\infty }{H^{M}\left(v\right)}^{2}dv=\vartheta^{2}\int_{0}^{\infty }e^{-{2\left(v-m\right)}^{2}/q}dv,$$

which can be written as follows:

$${\left\|H^{M}\left(v\right)\right\|}_{2}^{2}=\vartheta^{2}e^{-{2m}^{2}/q}\int_{0}^{\infty }e^{-{2v}^{2}/q+4vm/q}dv.$$

Therefore, using the known integral ([62] Equation (3.322.2)),

(A8) $$\int_{0}^{\infty }e^{-\frac{x^{2}}{4\beta }-\chi x}dx=\sqrt{\pi \beta }\;e^{{\beta \chi }^{2}}erfc\left(\chi \sqrt{\beta }\right)$$

and lying β=q/8 and χ=−4m/q, we immediately obtain

(A9) $${\left\|H^{M}\left(v\right)\right\|}_{2}^{2}=\vartheta^{2}\frac{\sqrt{\pi q/2}}{2}\;erfc\left(\frac{-m}{\sqrt{q/2}}\right),$$

whence and in view of the equality of the norms ${\left\|H^{M}\left(v\right)\right\|}_{2}={\left\|H\left(\tau \right)\right\|}_{2}$, the next equality follows

(A10) $${{\left\|H^{M}\left(v\right)\right\|}_{2}=\left\|H\left(\tau \right)\right\|}_{2}=\vartheta \frac{\sqrt[{4}]{\pi q/2}}{\sqrt{2}}\;\sqrt{erfc\left(\frac{-m}{\sqrt{q/2}}\right)}.$$

By (68), we have the following:

$${\left\|H\left(v\right)\right\|}_{2}^{2}=\int_{0}^{\infty }{H\left(v\right)}^{2}dv=\vartheta^{2}\int_{0}^{\infty }v^{2}e^{-2{\left(v-m\right)}^{2}/q}dv,$$

which, to facilitate determination of the integral, can be rewritten as follows

$${\left\|H\left(v\right)\right\|}_{2}^{2}=\vartheta^{2}e^{-2m^{2}/q}\int_{0}^{\infty }v^{2}e^{-2v^{2}/q+4vm/q}dv.$$

Whence, using the known integral ([62] Equation (3.462.7))

(A11) $$\int_{0}^{\infty }x^{2}e^{-\mu x^{2}-2\chi x}dx=-\frac{\chi }{2\mu^{2}}+\sqrt{\frac{\pi }{\mu^{5}}}\;\left(\frac{2\chi^{2}+\mu }{4}\right)e^{\chi^{2}/\mu }erfc\left(\frac{\chi }{\sqrt{\mu }}\right),$$

by lying μ=2/q and χ=−2m/q, we immediately obtain

(A12) $${\left\|H\left(v\right)\right\|}_{2}^{2}=\frac{1}{4}\vartheta^{2}\left[mq\;e^{-2m^{2}/q}+\sqrt{\frac{\pi q}{2}}\;\left(\frac{4m^{2}+q}{2}\right)erfc\left(\frac{-\sqrt{2}m}{\sqrt{q}}\right)\right],$$

whence

(A13) $${\left\|H\left(v\right)\right\|}_{2}=\frac{\vartheta }{2}\sqrt{\sqrt{\frac{q\pi }{2}}\left(\frac{{4m}^{2}+q}{2}\right)erfc\left(-\frac{\sqrt{2}\;m}{\sqrt{q}}\right)+mq{\;e}^{-2m^{2}/q}}.$$

### Appendix A.6. Norms of the Double-Mode Gauss Spectra $H\left(\tau \right)$ (70), $H\left(v\right)$ (71), and $H^{M}\left(v\right)$

We obtain the desired result presenting the spectrum $H\left(v\right)$ (71) as sum of two uni-mode Gauss spectra

(A14) $$H\left(v\right)=\vartheta_{1}ve^{-{\left(v-m_{1}\right)}^{2}/q_{1}}+\vartheta_{2}ve^{-{\left(v-m_{2}\right)}^{2}/q_{2}}=H_{1}\left(v\right)+H_{2}\left(v\right).$$

Therefore, we have the following:

(A15) $${\left\|H\left(v\right)\right\|}_{2}^{2}={\left\|H_{1}\left(v\right)\right\|}_{2}^{2}+{\left\|H_{2}\left(v\right)\right\|}_{2}^{2}+2\int_{0}^{\infty }H_{1}\left(v\right)H_{2}\left(v\right)dv,$$

where

$$\int_{0}^{\infty }H_{1}\left(v\right)H_{2}\left(v\right)dv=\vartheta_{1}\vartheta_{2}e^{-a}\int_{0}^{\infty }v^{2}e^{-\bar{q}v^{2}}e^{2v\bar{m}}dv,$$

with the parameters $\bar{m}$, $\bar{q}$ and a defined by the following formula:

(A16) $$\bar{m}=\frac{m_{1}}{q_{1}}+\frac{m_{2}}{q_{2}}\;\;,\;\bar{q}=\frac{1}{q_{1}}+\frac{1}{q_{2}}\;\;,\;a=\frac{m_{1}^{2}}{q_{1}}+\frac{m_{2}^{2}}{q_{2}}.$$

Therefore, by (A11), lying μ=2/q and χ=−2m/q, we immediately obtain

$$\int_{0}^{\infty }H_{1}\left(v\right)H_{2}\left(v\right)dv=\vartheta_{1}\vartheta_{2}e^{-a}\left[\frac{\bar{m}}{2{\bar{q}}^{2}}+\sqrt{\frac{\pi }{{\bar{q}}^{5}}}\;\left(\frac{2{\bar{m}}^{2}+\bar{q}}{4}\right)e^{{\bar{m}}^{2}/\bar{q}}erfc\left(-\frac{\bar{m}}{\sqrt{\bar{q}}}\right)\right],$$

which combined with Formula (A12) applied for $H_{1}\left(v\right)$ and $H_{2}\left(v\right)$, in view of (A15) yields

(A17) $${\left\|H\left(v\right)\right\|}_{2}^{2}=\frac{1}{4}\vartheta_{1}^{2}\left[m_{1}q_{1}\;e^{-2m_{1}^{2}/q_{1}}+\sqrt{\frac{\pi q_{1}}{2}}\;\left(\frac{4m_{1}^{2}+q_{1}}{2}\right)erfc\left(\frac{-\sqrt{2}m_{1}}{\sqrt{q_{1}}}\right)\right]+\;\frac{1}{4}\vartheta_{2}^{2}\left[m_{2}q_{2}\;e^{-2m_{2}^{2}/q_{2}}+\sqrt{\frac{\pi q_{2}}{2}}\;\left(\frac{4m_{2}^{2}+q_{2}}{2}\right)erfc\left(\frac{-\sqrt{2}m_{2}}{\sqrt{q_{2}}}\right)\right]+\;2\vartheta_{1}\vartheta_{2}e^{-a}\left[\frac{\bar{m}}{2{\bar{q}}^{2}}+\sqrt{\frac{\pi }{{\bar{q}}^{5}}}\;\left(\frac{2{\bar{m}}^{2}+\bar{q}}{4}\right)e^{{\bar{m}}^{2}/\bar{q}}erfc\left(-\frac{\bar{m}}{\sqrt{\bar{q}}}\right)\right].$$

By (A14) and (4)

$$H^{M}\left(v\right)=\vartheta_{1}e^{-{\left(v-m_{1}\right)}^{2}/q_{1}}+\vartheta_{2}e^{-{\left(v-m_{2}\right)}^{2}/q_{2}}=H_{1}^{M}\left(v\right)+H_{2}^{M}\left(v\right).$$

Therefore, as above,

(A18) $${\left\|H^{M}\left(v\right)\right\|}_{2}^{2}={\left\|H_{1}^{M}\left(v\right)\right\|}_{2}^{2}+{\left\|H_{2}^{M}\left(v\right)\right\|}_{2}^{2}+2\int_{0}^{\infty }H_{1}^{M}\left(v\right)H_{2}^{M}\left(v\right)dv,$$

where

$$\int_{0}^{\infty }H_{1}^{M}\left(v\right)H_{2}^{M}\left(v\right)dv=\vartheta_{1}\vartheta_{2}e^{-a}\int_{0}^{\infty }e^{-\bar{q}v^{2}}e^{2v\bar{m}}d\tau ,$$

with the parameters $\bar{m}$, $\bar{q}$ and a defined by (A16), which, by Equation (A8), lying $\beta =\frac{1}{4\bar{q}}$ and $\chi =-2\bar{m}$, can be expresses as follows:

$$\int_{0}^{\infty }H_{1}^{M}\left(v\right)H_{2}^{M}\left(v\right)dv=\frac{1}{2}\vartheta_{1}\vartheta_{2}e^{-a}\sqrt{\frac{\pi }{\bar{q}}}\;e^{\frac{{\bar{m}}^{2}}{\bar{q}}}erfc\left(-\bar{m}\sqrt{\frac{1}{\bar{q}}}\right).$$

Substituting the above into (A18) and combining with (A9) applied for the partial spectra, $H_{1}^{M}\left(v\right)$ and $H_{2}^{M}\left(v\right)$, we obtain

$${\left\|H^{M}\left(v\right)\right\|}_{2}^{2}=\vartheta_{1}^{2}\frac{\sqrt{\pi q_{1}/2}}{2}\;erfc\left(\frac{-m_{1}}{\sqrt{q_{1}/2}}\right)+\vartheta_{2}^{2}\frac{\sqrt{\pi q_{2}/2}}{2}\;erfc\left(\frac{-m_{2}}{\sqrt{q_{2}/2}}\right)+\;\vartheta_{1}\vartheta_{2}e^{-a}\sqrt{\frac{\pi }{\bar{q}}}\;e^{\frac{{\bar{m}}^{2}}{\bar{q}}}erfc\left(-\bar{m}\sqrt{\frac{1}{\bar{q}}}\right),$$

whence, for ${\left\|H\left(\tau \right)\right\|}_{2}={\left\|H^{M}\left(v\right)\right\|}_{2}$, the next formula follows

(A19) $${\left\|H\left(\tau \right)\right\|}_{2}={\left[\frac{\sqrt{\pi q_{1}/2}{\;\vartheta }_{1}^{2}}{2}\;erfc\left(\frac{-m_{1}}{\sqrt{q_{1}/2}}\right)+\frac{\sqrt{\pi q_{2}/2\;}\vartheta_{2}^{2}}{2}\;erfc\left(\frac{-m_{2}}{\sqrt{q_{2}/2}}\right)+\vartheta_{1}\vartheta_{2}\sqrt{\frac{\pi }{\bar{q}}}\;e^{\frac{{\bar{m}}^{2}}{\bar{q}}-a}erfc\left(-\frac{\bar{m}}{\sqrt{\bar{q}}}\right)\right]}^{\frac{1}{2}}$$
